# A sequential multimodal framework for spinal cord regeneration

**DOI:** 10.3389/fncel.2026.1790692

**Published:** 2026-04-29

**Authors:** Eduardo Blat Sos

**Affiliations:** Universidad CEU Cardenal Herrera, Valencia, Spain

**Keywords:** nervous system, neurobiology, neurology, neuropathology, neuroscience, neurosurgery, spinal cord, spine injury

## Abstract

This hypothetical study explores a potential therapeutic strategy for patients with recent complete spinal cord injuries (≤6 weeks since injury approximately). We review current literature and identify five key determinants of regenerative failure: inflammation, glial scar formation, deficit in molecular guidance, loss of structural guidance, and persistence of Wallerian debris. Although each of these barriers can be addressed by existing interventions, their efficacy depends partially on a constrained sequence of application. Based on this, a temporally orchestrated strategy is proposed, comprising early immunomodulation to stabilize the lesion environment, mechanical realignment combined with implantation of a temporary extracellular matrix to restore structural continuity, sustained molecular guidance and matrix remodeling to guide axonal growth. The proposed framework offers a mechanistically integrated and testable hypothesis for promoting functional spinal cord regeneration.

## 1 Introduction

Spinal cord injuries (SCI), part of the central nervous system (CNS), represent one of the most significant health challenges in modern society, with an estimated 250,000 to 500,000 new cases worldwide each year. These injuries are highly debilitating, often reducing both life expectancy and functional capacity in affected individuals.

Unlike the peripheral nervous system (PNS), which exhibits efficient regenerative capacity, the adult CNS faces cellular and molecular barriers that prevent effective axonal elongation after injury. These barriers include changes in the extracellular matrix, the formation of reactive glia, and the presence of inhibitory signals such as Nogo-A, MAG, and OMgp (Bradbury and Burnside, 2019).

Studies, including those by Schwab and Strittmatter (2014), have demonstrated that under permissive conditions, CNS axons are capable of regeneration. This suggests that the primary limitation is not an intrinsic inability of neurons, but rather the inhibitory environment that forms after injury. These observations have motivated strategies aimed at modifying the post-injury environment to promote axonal growth.

In this context, we propose the induction of a temporary extracellular matrix around the injured axonal surface to support regeneration, combined with targeted modulation of the cellular environment. This matrix would consist of type I collagen, RGD motifs, and laminin. Furthermore, we propose the strategic incorporation of chemorepellent and chemoattractant cues to guide axonal growth.

## 2 Theoretical framework

In humans, the nervous system is divided into two main components: the central nervous system (CNS) and the peripheral nervous system (PNS). One of the most notable differences between these systems lies in their response to injury. Unlike the PNS, which exhibits robust regenerative capacity following damage, the CNS demonstrates a limited regenerative response, hindering functional recovery. This divergence in regenerative potential between the CNS and PNS is a central topic in spinal cord injury research and is fundamental for understanding the biological mechanisms underlying neural repair.

### 2.1 Spinal cord organization

**FIGURE 1:**
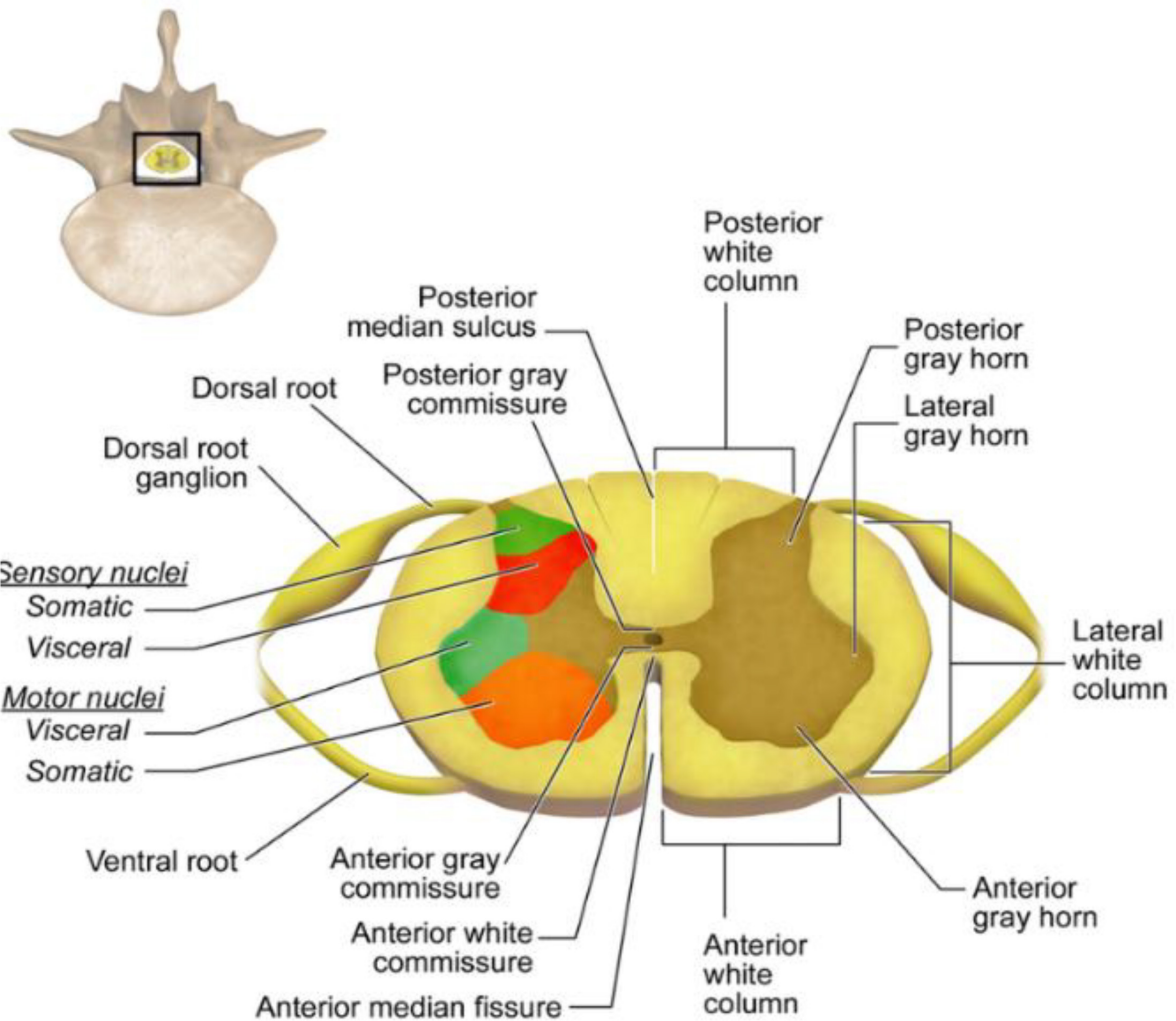
Transverse section of the spinal cord showing dorsal and ventral roots, white matter columns, gray matter horns, and motor and sensory nuclei. Adapted from Bruce Blaus, Wikimedia Commons, licensed under CC BY-SA 4.0.

The spinal cord is a critical structure of the central nervous system (CNS), serving as the primary conduit between the brain and the body. As shown in Figure 1, it is organized into dorsal horns, which receive sensory information, and ventral horns, which transmit motor signals. Surrounding gray matter, the white matter consists of myelinated axons organized into ascending (sensory) and descending (motor) tracts, which are essential for normal spinal cord function.

#### 2.1.1 Reparation system in PNS

**FIGURE 2:**
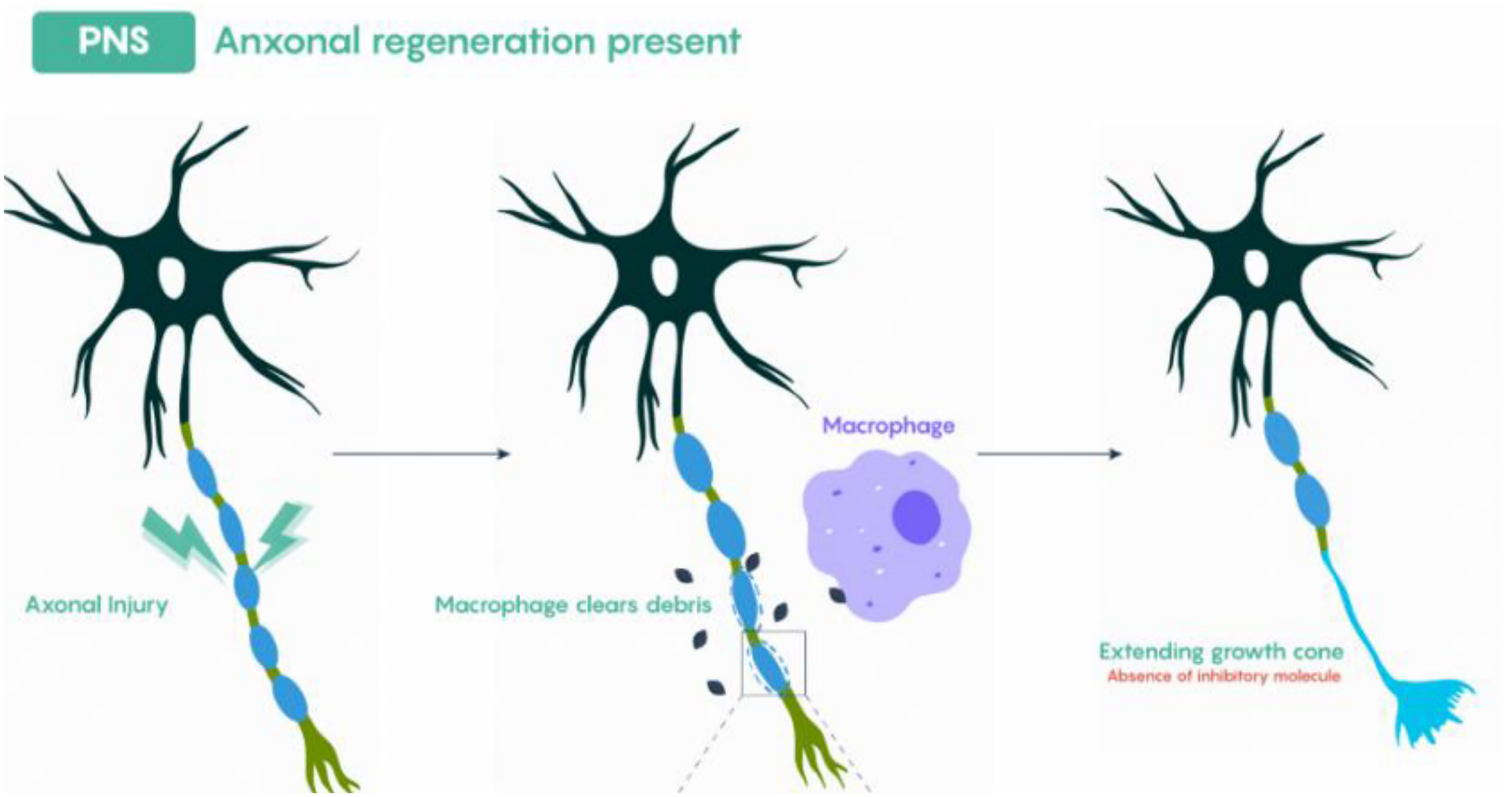
General scheme of axonal regeneration in the PNS. Adapted from Neuroscience: Canadian 3rd Edition. Image under Creative Commons license.

As shown in Figure 2, following a peripheral nerve injury, the segment of the axon distal to the lesion undergoes Wallerian degeneration, a highly coordinated process involving the fragmentation of the axonal cytoskeleton and disassembly of myelin sheaths. Debris from the axon and myelin are actively cleared by Schwann cells and infiltrating macrophages (Rotshenker, 2011). Schwann cells, which previously maintained the myelin sheath, dedifferentiate into a repair-promoting phenotype. This phenotypic transition is tightly regulated at the transcriptional level: upregulation of c-Jun and Sox2 signaling represses myelin-specific genes while activating those involved in proliferation, extracellular matrix (ECM) remodeling, and neurotrophic factor production (Arthur-Farraj et al., 2012). Intracellular pathways, including MAPK/ERK, PI3K/Akt, and JNK, are central to orchestrating this shift, enabling Schwann cells to coordinate cytoskeletal reorganization, migration, and secretion of regenerative molecules.

Dedifferentiated Schwann cells proliferate rapidly and align along the basal lamina to form Büngner bands. These structures act as physical and biochemical conduits for regenerating axons. Büngner bands are rich in neurotrophic factors such as nerve growth factor (NGF), brain-derived neurotrophic factor (BDNF), glial cell line-derived neurotrophic factor (GDNF), and neurotrophin-3 (NT-3), which activate Trk receptors on axons to promote survival and directional growth (Allodi et al., 2012).

Simultaneously, cell adhesion molecules, including laminin, fibronectin, N-cadherin, and integrins, provide adhesive substrates for axonal elongation. Schwann cells also secrete chemoattractants, such as CXCL12 and CCL2, forming chemotactic gradients that guide regenerating axons toward their targets. Conversely, chemorepellent molecules, including semaphorin 3A, ephrin-A, and Slit proteins, are expressed in defined spatial patterns to prevent misguided branching or reinnervation of inappropriate regions (Allodi et al., 2012). The balance of chemoattractant and chemorepellent signaling ensures precise axonal navigation through the extracellular environment.

In addition to these guidance cues, Schwann cells respond dynamically to damage-associated molecular patterns (DAMPs) released from degenerating axons. Molecules such as ATP, HMGB1, and heat shock proteins bind to pattern recognition receptors (PRRs) on Schwann cells, triggering downstream signaling via NF-κB, MAPK, and inflammasome pathways (Jessen and Mirsky, 2016). These signals regulate the secretion of pro-regenerative cytokines, chemokines, and growth factors, while modulating the recruitment and activation of macrophages for efficient debris clearance. The integration of DAMP signals with neurotrophic and chemotactic cues enables Schwann cells to coordinate inflammation, axonal guidance, and ECM remodeling in a spatially and temporally precise manner.

**FIGURE 3:**
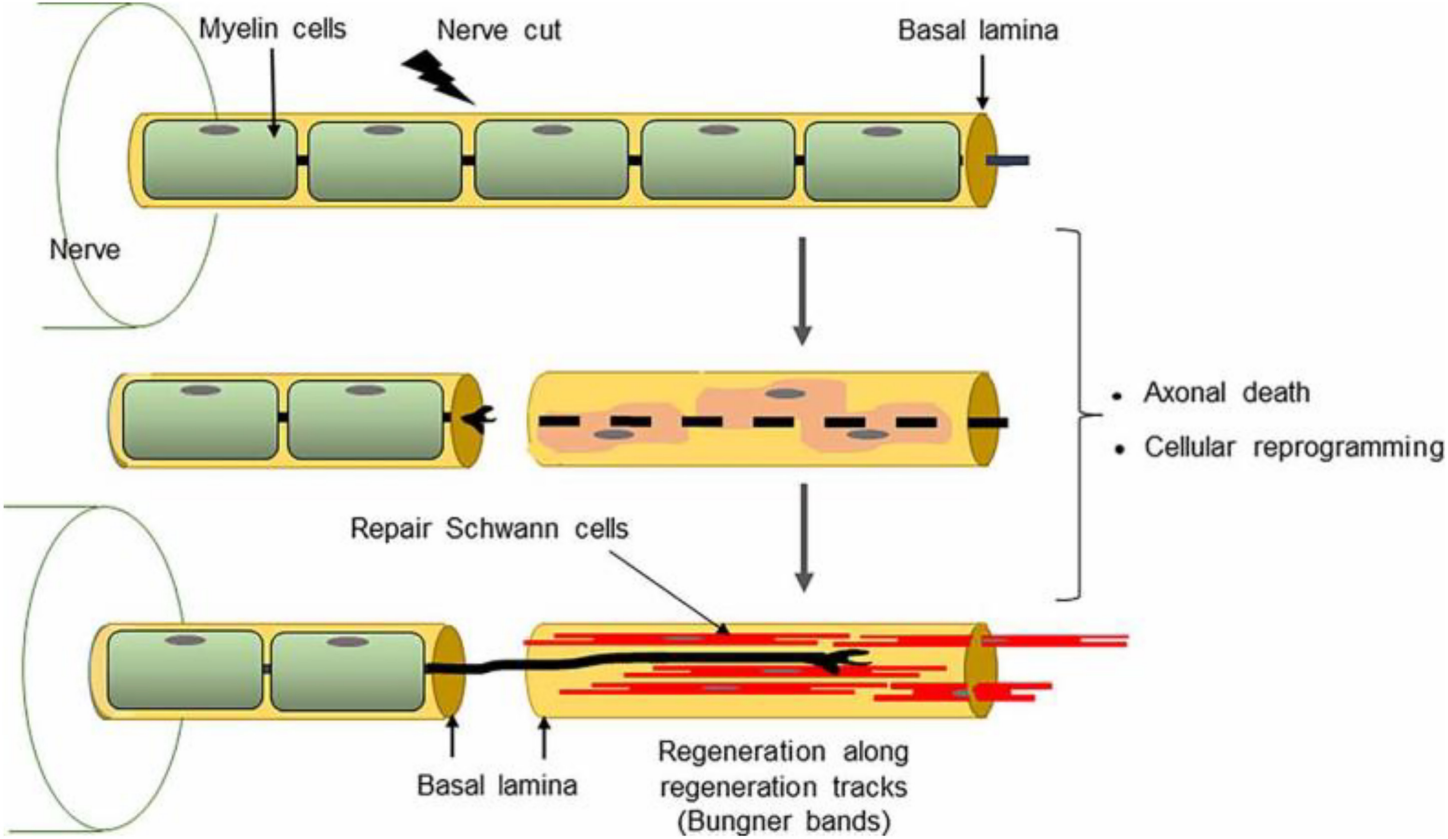
Schematic representation of peripheral nerve regeneration, highlighting the role of Büngner bands. Adapted from Arthur-Farraj, P., et al. (2012), The Role of c-Jun and Autocrine Signaling Loops in the Control of Repair Schwann Cells and Regeneration, Journal of Cell Biology. Image reused under CC BY 4.0.

As shown in Figure 3, regenerating axons encounter the Büngner bands and they extend along these scaffolds, guided by gradients of neurotrophic factors and adhesion molecules. Schwann cells maintain direct physical contact with axons via N-cadherin-mediated adhesion and integrin signaling, transmitting mechanical and biochemical signals that stabilize growth cones and promote directional elongation. Once axons reach their targets, Schwann cells redifferentiate into a myelinating phenotype. This involves downregulation of c-Jun and Sox2, reactivation of myelin-specific transcription factors, and the assembly of compact myelin sheaths (Nocera and Jacob, 2020). Proper remyelination restores saltatory conduction, metabolic support, and functional connectivity of the regenerated nerve.

#### 2.1.2 CNS reparation system

**FIGURE 4:**
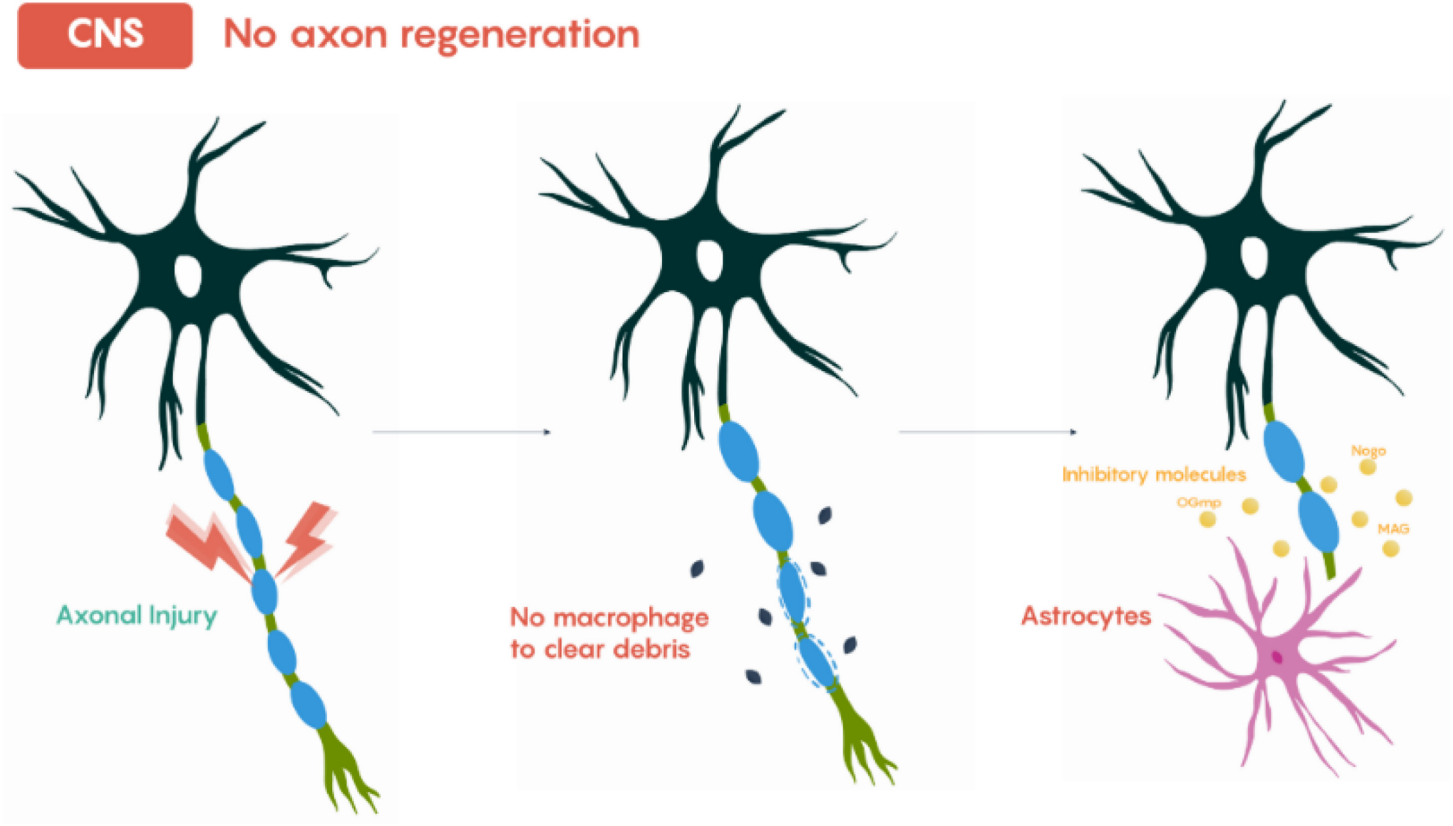
General scheme of the post-axonal injury phases in the CNS. Adapted from Neuroscience: Canadian 3rd Edition. Image under Creative Commons license.

**FIGURE 5:**
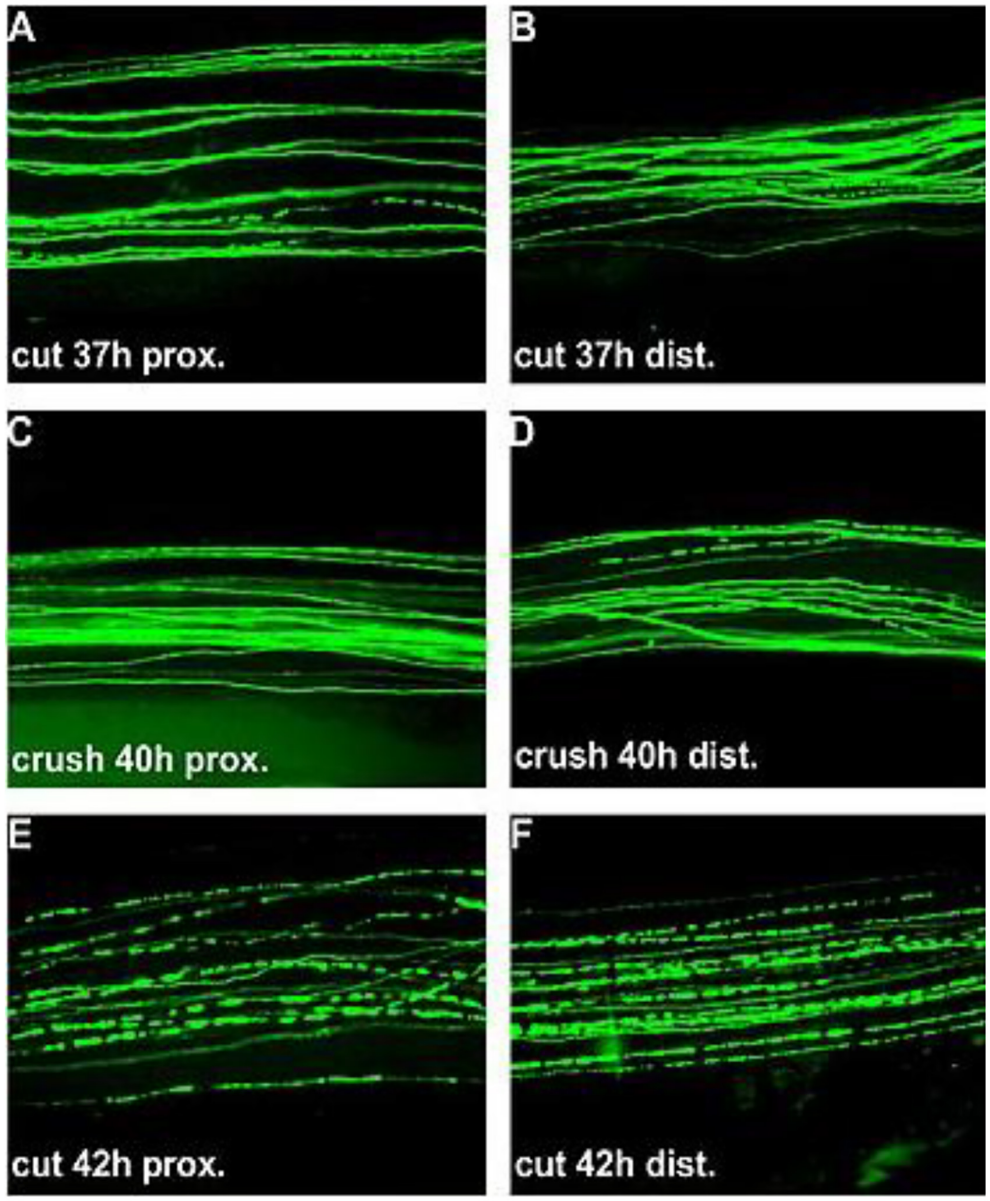
Wallerian degeneration via fluorescence from 37 h **(A)** to 42 h **(F)**. Bogdan Beirowski. Wikipedia Commons. Licensed under Creative Commons Attribution 2.0 International.

Regeneration following injury in the central nervous system (CNS) is markedly more limited than in the peripheral nervous system (PNS), as shown in Figure 4, due to both intrinsic neuronal factors and extrinsic inhibitory environments. After CNS axonal injury, Wallerian degeneration, as shown in Figure 5 still occurs in the distal axon segments; however, the clearance of myelin debris is slower and less efficient than in the PNS. Oligodendrocytes, the CNS myelinating cells, do not dedifferentiate into a repair-promoting phenotype comparable to Schwann cells, nor do they form guidance structures analogous to Büngner bands (Yiu and He, 2006). Consequently, regenerating CNS axons lack both a permissive scaffold and the coordinated molecular signals that Schwann cells provide in the PNS.

At the molecular level, CNS myelin expresses potent axon growth inhibitors, including Nogo-A, myelin-associated glycoprotein (MAG), and oligodendrocyte myelin glycoprotein (OMgp). These molecules interact with neuronal receptors such as Nogo receptor 1 (NgR1), paired immunoglobulin-like receptor B (PirB), activating intracellular signaling pathways like RhoA/ROCK, PTEN/mTOR suppression. The result is growth cone collapse, cytoskeletal stabilization against elongation, and suppression of axonal regeneration (Schwab, 2004). In contrast, Schwann cells in the PNS actively degrade inhibitory molecules through proteolytic enzymes, secrete neurotrophic factors such as NGF, BDNF, and GDNF, and organize Büngner bands along the basal lamina, providing both biochemical and structural guidance for axonal extension.

A major extrinsic barrier in the CNS is glial scar formation, which is composed of reactive astrocytes, microglia, oligodendrocyte precursor cells, and an extracellular matrix enriched in chondroitin sulfate proteoglycans (CSPGs) such as brevican, neurocan, versican, and aggrecan. CSPGs inhibit axonal growth by binding to receptors like protein tyrosine phosphatase sigma (PTPσ) and NgR1, triggering intracellular pathways including RhoA/ROCK and downstream cytoskeletal collapse (Fawcett and Asher, 1999). Furthermore, reactive astrocytes secrete inhibitory cytokines, including TGF-β and endothelin-1, which reinforce scar formation and restrict regenerative sprouting.

CNS neurons themselves also display a reduced intrinsic regenerative capacity. Expression of transcription factors promoting axonal growth, such as c-Jun, Sox11, and KLF family members, is often limited after injury, whereas PNS neurons can rapidly upregulate these factors to stimulate cytoskeletal reorganization, axonal transport, and growth cone formation (Moore and Goldberg, 2011). Additionally, CNS axons are more vulnerable to oxidative stress and excitotoxicity due to insufficient metabolic and trophic support after injury, further limiting regeneration.

Taken together, the biochemical and structural environment of the CNS is inherently non-permissive. The absence of Büngner-like guidance bands, persistence of myelin-derived inhibitors, formation of inhibitory glial scars rich in CSPGs, and limited intrinsic growth programs converge to produce minimal axonal regrowth. By contrast, the PNS combines dedifferentiated Schwann cells, guided Büngner bands, neurotrophic factor secretion, chemoattractant gradients, and DAMP-mediated regenerative signaling to create a permissive, coordinated environment that supports effective axonal extension and reconnection. This dichotomy highlights why CNS regeneration remains a major challenge and why strategies to mimic PNS-like conditions (through CSPG degradation, neutralization of myelin inhibitors, or Schwann cell transplantation) are central to experimental CNS repair approaches (Thuret et al., 2006).

### 2.2 Conceptual framework: from individual barriers to integrated solutions

The structure of this investigation is built upon a logical progression that addresses the current regenerative failure in the central nervous system (CNS) through a three-pillared dialectic. The first pillar dissects the specific mechanisms that produce regenerative failure; the second evaluates whether each mechanism can be resolved by an available therapeutic strategy; and the third integrates these findings into a unified, falsifiable hypothesis. Each pillar constitutes a necessary precondition for the next, converting the analysis from a literature review into a scientific argument:

#### 2.2.1 Determinants of regenerative failure in acute axonal injury

The first pillar requires a dissection of specific mechanisms that prevent axonal regeneration following recent trauma. Current literature identifies five critical factors whose convergence creates a hostile environment for neurite outgrowth:

1. Glial scar formation: Reactive astrocytes, microglia, and oligodendrocyte precursor cells consolidate a physical and biochemical barrier enriched in chondroitin sulfate proteoglycans (CSPGs), which bind to axonal receptors (PTPσ, NgR1) and activate RhoA/ROCK-mediated growth cone collapse (Bradbury and Burnside, 2019; Fawcett and Asher, 1999).
2. Generalized inflammation: Damage-associated molecular patterns (DAMPs) released from necrotic cells (including ATP, HMGB1, and heat shock proteins) activate innate immune signaling in microglia, astrocytes, and infiltrating macrophages, generating a pro-inflammatory milieu that sustains secondary damage (Rotshenker, 2011).
3. Deficit in molecular guidance: The lack of coordinated biochemical signals to attract or repel growth cones (Allodi et al., 2012).
4. Loss of structural guidance: The CNS lacks the anatomical equivalent of Büngner bands. Oligodendrocytes do not dedifferentiate into a repair phenotype, and no basal lamina scaffold is reconstituted to provide contact guidance for elongating neurites (Jessen and Mirsky, 2016; Yiu and He, 2006).
5. Wallerian debris persistence: Following axonal injury, degenerating axons and myelin generate a complex pool of cellular and extracellular debris. In early stages, this material may transiently contribute to scaffold incorporation by providing residual structural elements and molecular anchoring points. However, its persistence becomes progressively detrimental, as myelin-associated inhibitors and disorganized remnants of axonal architecture interfere with growth cone progression and disrupt directed neurite extension, ultimately constituting a physical and biochemical barrier to regeneration (Schwab and Strittmatter, 2014; Schwab, 2004).
6. Time after injury: Although often treated as an independent determinant, the post-traumatic interval is not a causal factor *per se* but rather a proxy for the underlying time-dependent biological processes that govern regenerative capacity. As time progresses, the lesion environment transitions from a transiently permissive state to a chronically inhibitory one. Evidence from human spinal cord histopathology shows that during the subacute phase, axonal elements and peri-axonal proteins are progressively lost while inhibitory myelin debris persists. By the end of this period, the structural substrates required for reconnection are largely dismantled, marking a practical upper limit for effective regenerative intervention. Most studies define the end of the subacute phase at approximately 6 weeks post-injury. (Becerra et al., 1995)

These six factors do not operate independently. The inflammatory microenvironment accelerates Wallerian degeneration, which in turn compounds the loss of structural substrates and reduces integrin density on axonal surfaces. Glial scar formation is partly driven by the same DAMP signals that perpetuate inflammation. The temporal constraint is therefore not a sixth independent barrier but a consequence of the other five acting together. Therefore, these constraints implicitly define a non-arbitrary, biologically determined sequence of intervention. Because each factor causally amplifies the others, attempting to address them out of order would not only reduce efficacy but may actively counteract the therapeutic objective.

In this sense, the therapeutic logic is not imposed externally but emerges directly from the intrinsic dependencies between the pathological processes. The order of intervention is therefore pre-defined by the system’s own dynamics, requiring a sequential, interdependent approach rather than parallel or isolated strategies.

By this logic, a regressive deduction scheme is formed:


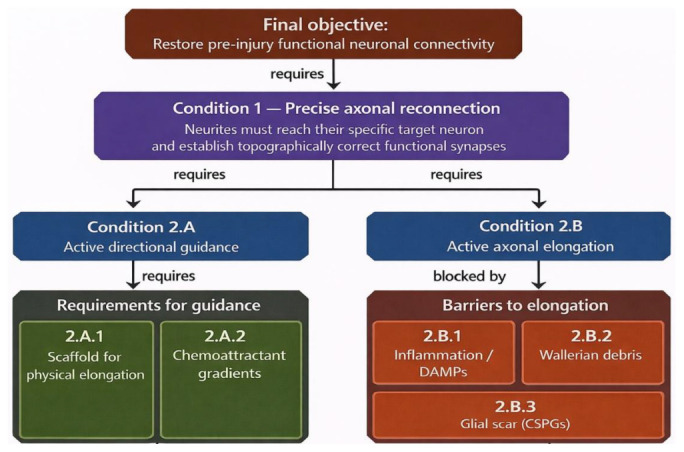


#### 2.2.2 Evaluation of individual therapeutic viability

The previous section established that regenerative failure in acute spinal cord injury is not the product of a single mechanism but of the convergence of five distinct barriers. Identifying these barriers, however, is only the first step. The central question that this section addresses is whether each barrier can be resolved by an available therapeutic strategy, and whether those strategies can be integrated into a coherent, sequential protocol without mutual interference.

To answer this question, we adopt a dialectical and inductive method. For each barrier, we pose a precise clinical question, survey the available interventions supported by preclinical or clinical evidence, compare them across mechanistic, pharmacokinetic, and compatibility criteria, and arrive at a reasoned selection. The final hypothesis is presented in Section 3 as the synthesis of the argument constructed here.

Study selection criteria:

Studies were considered for inclusion if they met the following criteria:

1. The intervention targeted at least one of the five identified barriers to CNS axonal regeneration, inflammation and DAMP signaling, loss of structural guidance, deficit in molecular guidance, glial scar formation, or Wallerian debris persistence
2. The study reported quantifiable outcomes directly relevant to axonal regeneration, including but not limited to neurite outgrowth length, locomotor recovery scores, histological evidence of axonal sprouting, CSPG degradation, or macrophage phenotype ratios;
3. The intervention was delivered in an *in vivo* spinal cord injury model or, where *in vivo* data were unavailable, in a validated *in vitro* system using primary neurons or dorsal root ganglion explants under conditions representative of the post-injury microenvironment
4. The study provided sufficient mechanistic or pharmacokinetic detail to permit comparison across the selection criteria applied here.

Studies were excluded if they addressed chronic SCI exclusively, defined as injury interval exceeding 6 weeks post-trauma, as the biological substrate for the proposed interventions (preserved axonal surface integrins, residual ECM anchoring points, and a transiently permissive inflammatory window) is no longer available beyond this threshold. Studies were further excluded if their primary outcome was neuroprotection without a regenerative component. No language or publication date restrictions were applied; however, given the mechanistic focus of the selection process, priority was assigned to studies providing quantitative dose-response, pharmacokinetic, or direct comparative data over qualitative or purely descriptive reports.

##### 2.2.2.1 Inflammation and DAMP signaling

Clinical question: Can the acute inflammatory microenvironment be attenuated sufficiently to permit subsequent regenerative interventions without suppressing endogenous repair mechanisms?

The inflammatory cascade initiated by DAMPs is a principal driver of secondary injury in SCI. Therapeutic strategies targeting this cascade include systemic corticosteroids, systemic cytokine administration, and locally delivered biomaterial systems. The table below compares five key studies addressing this barrier.

**Table:** 

Study	Model	Intervention	Key finding
Shen et al., 2022	Rat SCI	DAMP-scavenging + IL-10 hydrogel	Sustained IL-10 release reduced TNF-α and IL-1β; promoted M2 macrophage polarization and axonal sprouting at the lesion.
Bethea et al., 1999	Rat SCI	Systemic IL-10 (intravenous)	IL-10 reduced TNF-α production and significantly improved hindlimb motor scores at 6 weeks.
Bracken et al., 1997	Human RCT	Methylprednisolone (24–48 h IV)	Modest motor improvement when given ≤8 h post-injury; significant immunosuppression side-effects with prolonged use.
Fehlings et al., 2012	Rat SCI	Early surgical decompression + cytokine inhibition	Early decompression combined with cytokine suppression accelerated neurological recovery vs. decompression alone.
David and Lacroix, 2003	Mouse SCI	Genetic deletion of TNF-α/IL-1β	Partial reduction of secondary lesion size; macrophage infiltration persisted, indicating redundant inflammatory pathways.

Analysis of these five studies reveals a convergent conclusion: anti-inflammatory intervention is most effective when it is: (1) Delivered locally rather than systemically. (2) Sustained over the acute-to-subacute transition. (3) Capable of targeting both soluble DAMP signals and downstream cytokine cascades simultaneously.

Single-cytokine blockade (David and Lacroix, 2003) is insufficient because of pathway redundancy, while broad systemic immunosuppression (Bracken et al., 1997) produces unacceptable side-effects and blunts endogenous repair signals.

Quantitative conclusion: why Shen et al. (2022) is the superior strategy

The selection of Shen et al. is driven by three quantitative arguments that the other four studies collectively establish.

First, the ceiling of systemic approaches. Bracken et al. (1997), the largest dataset in the table at 487 patients, produces a null result in its primary analysis (ASIA motor score improvement *p* = 0.43 vs. placebo in the most recent pooled re-analysis of NASCIS-II and Sygen), with sepsis and pneumonia incidence consistently higher in the methylprednisolone groups. The only evidence of benefit is a *post hoc* subgroup, which contemporary statistical standards do not accept as confirmatory. Bethea et al. confirms IL-10 reduces TNF-α and improves motor scores in rats, but with a plasma half-life of 2–4 h and no spatial confinement, sustained effect requires repeated systemic dosing, introducing the same accumulation and immunosuppression risks that disqualify methylprednisolone.

Second, the ceiling of cytokine-level blockade. David and Lacroix (2003) establishes this most clearly: complete genetic elimination of both TNF-α and IL-1β, more thorough than any pharmacological approach, still left macrophage infiltration intact, because NLRP3 inflammasome (ATP), RAGE (HMGB1), and TLR4 pathways continue driving M1 polarization independently. Any strategy targeting cytokines downstream of DAMPs cannot resolve this redundancy.

Third, Shen et al. (2022) is the only study in the table with statistically significant superiority across both inflammatory biomarkers and motor function relative to all internal comparators simultaneously: TNF-α and IL-1β significantly lower than all three control groups at day 3 (*p* < 0.05), highest M2/M1 ratio at day 7, and highest BMS locomotor score at 8 weeks in complete transection, all within a single four-group design that isolates each component’s contribution. IL-10 loading at 12.5 μg mL^–1^ delivers approximately 60% within the first 24 h, covering the peak DAMP release window, with a 7-day sustained tail requiring no re-dosing. No other strategy in this table satisfies all three protocol requirements (local delivery, temporal self-limitation, and upstream DAMP targeting) with direct quantitative evidence.

##### 2.2.2.2 Loss of structural guidance

The analysis for this section proceeds in three steps, each following from the previous. The first establishes what properties a scaffold matrix must have given the biology of the injured spinal cord. The second shows which material satisfies those properties and why the alternatives do not. The third shows that even the best single material is insufficient and derives the additional components from that insufficiency rather than asserting them.

Step 1: What does the injured axonal environment demand from a scaffold?

After acute SCI, the lesion environment presents a specific set of physical and molecular conditions that define what a scaffold must do. Three functional requirements emerge from the literature, and together they constitute the filter through which all candidate materials must pass.

First, the scaffold must provide directional information to elongating growth cones. Regenerating axons do not extend randomly, they follow physical topography through a process known as contact guidance, in which growth cone mechanosensing at the neurite tip detects and responds to the local fibrillar architecture of the extracellular environment. Dubey et al. (1999) demonstrated this directly: neurite elongation from dorsal root ganglion explants into magnetically aligned collagen gel rods was substantially greater than in unaligned controls and increased progressively with magnetic field strength as collagen fibril alignment along the rod axis became more pronounced. Critically, the axial bias of neurite elongation mirrored the degree of fibrillar organization, confirming that growth cones follow physical structure rather than chemical gradients alone. An amorphous hydrogel without organized fibrillar architecture cannot provide this guidance. The scaffold must therefore possess inherent fibrillar structure at the spatial scale detectable by growth cone mechanosensing, a scale that collagen, by virtue of its native fibrillogenesis, naturally occupies.

Second, the scaffold must be deliverable to an irregular, fluid-filled lesion cavity without requiring open reconstructive surgery. The post-transection cavity is not a geometric void, but a dynamic, heterogeneous space bounded by reactive tissue, residual debris, and variable perfusion. A rigid preformed scaffold cannot conform to this environment. Zweckberger et al. (2016) demonstrated that self-assembling injectable scaffolds can optimize the post-traumatic milieu and synergistically enhance the effects of neural stem cell therapy after cervical SCI, establishing injectability not merely as a convenience but as a functional requirement for achieving uniform lesion fill, scaffold-tissue integration, and homogeneous delivery of therapeutic cargo.

Third, and critically for this protocol, the scaffold must carry and release bioactive agents without inactivating them and must do so from a material that is biochemically neutral with respect to those agents. Han et al. (2009) showed that linear ordered collagen scaffolds loaded with collagen-binding neurotrophin-3 promoted axonal regeneration and partial functional recovery after complete spinal cord transection, establishing that collagen can be functionalized through physical absorption or immobilization of bioactive proteins that release continuously from the matrix. This positions collagen not as a passive substrate but as an active delivery vehicle whose fibrillar architecture simultaneously guides axons and presents molecular cues in a spatially organized manner.

Step 2: Which material passes the filter, and why do the alternatives fail?

The candidate materials are type I collagen, fibrin, hyaluronic acid, matrigel, and synthetic hydrogels. Each fails at least one of the three criteria established in Step 1.

Matrigel is immediately excluded on bioactive vehicle grounds: its composition is undefined and batch-variable, meaning it cannot be reliably co-formulated with specific agents at controlled concentrations, and its high endogenous growth factor content would interfere with the precise delivery of exogenous therapeutic molecules (Kleinman and Martin, 2005).

Synthetic hydrogels (PEG, PLGA) satisfy the injectable criterion and offer mechanical tunability, but they lack fibrillar architecture — their crosslinked network is isotropic at the nanoscale and provides no topographic guidance signal to growth cones. They also produce degradation byproducts (lactic acid from PLGA, acidic oligomers from PEG esters) that shift local pH away from the optimal range required for ChABC enzymatic activity, compromising the biochemical neutrality criterion (Führmann et al., 2017).

Hyaluronic acid is present in neural ECM and supports cell migration, but it is naturally amorphous and therefore provides no contact guidance. Direct experimental comparison of HA, laminin, fibronectin, and collagen I confirmed that HA concentration did not have significant effects on neurite extension, establishing that its contribution to directional axonal growth is negligible when isolated from other matrix components (Tian et al., 2005).

Fibrin has fibrillar structure and is biologically active, but its primary limitation is degradation rate. Fibrin is cleaved by plasmin within 24–72 h in the post-injury environment, where plasminogen activators are markedly elevated following SCI. This is substantially faster than the axonal elongation timeline requires, and premature scaffold dissolution would leave regenerating axons without structural support before they have traversed the lesion gap (Sakiyama-Elbert et al., 2001).

Type I collagen satisfies all three criteria simultaneously. It is injectable and crosslinks through self-assembly at physiological temperature without toxic chemical initiators. Its fibrillar architecture — triple-helical monomers assembling into 50–200 nm diameter fibrils at the scale of growth cone filopodia — provides the topographic signal that the contact guidance literature demonstrates is necessary for directional elongation (Dubey et al., 1999). It is a proven bioactive vehicle: collagen scaffolds can be functionalized by physical absorption or immobilization of bioactive proteins that release continuously from the matrix without loss of biological activity (Han et al., 2009). Furthermore, its mechanical stiffness is directly tunable by concentration, a parameter with direct experimental validation in the axonal growth context. Gel stiffness increased significantly with collagen concentration, ranging from 2.2 Pa at 0.4 mg/mL to 17.0 Pa at 2.0 mg/mL, and maximum neurite extension from dissociated chick dorsal root ganglia was observed in lower concentration gels of 0.6–0.8 mg/mL, with neurite length increasing across all gels between day 1 and day 4 except at the highest concentration tested, where a decrease was noted at day 4. This establishes a quantitative inverse relationship between scaffold stiffness and axonal outgrowth that no other candidate material in this comparison offers with equivalent experimental resolution (Willits and Skornia, 2004).Finally, type I collagen has been shown to support axonal regeneration and functional recovery when implanted as an oriented scaffold in complete spinal cord transection models, providing *in vivo* validation beyond the *in vitro* contact guidance data (Yoshii et al., 2003).

The convergence of injectability, fibrillar topography, bioactive vehicle capacity, and concentration-dependent mechanical tunability makes type I collagen the only material in the candidate set that passes all three filters without requiring chemical modification that would introduce secondary risks in the CNS environment. Collagen I is therefore selected as the structural base of the TEM. But this selection immediately reveals a problem.

Step 3: What collagen I cannot do and what that demands

Collagen I’s adhesion to axonal membranes depends on α1β1 and α2β1 integrin pairs, which bind the GFOGER motif in the triple-helical collagen domain. However, during Wallerian degeneration, progressive breakdown of the axonal membrane systematically reduces integrin surface expression, including these receptors. A collagen I scaffold therefore presents its adhesion domains to a membrane with diminishing functional α1β1/α2β1 availability as post-lesion degeneration advances. This is not a failure of the material, it is a structural consequence of the injury biology that demands compensation through parallel adhesion systems (Plantman et al., 2008).

Laminin addresses this gap directly. Among ECM proteins tested on adult DRG neurons, laminins were by far the most active molecules for neurite outgrowth, engaging α3β1, α6β1, and α7β1 integrins — receptor families distinct from that collagen I occupies (Plantman et al., 2008). These pathways also activate PI3K/Akt survival signaling, providing neuroprotection beyond simple adhesion. Quantitatively, a co-gel of collagen I and laminin produced an average neurite length of 1532 ± 91 versus 976 ± 32 μm for controls, representing an 85.9% ± 9.3% increase in outgrowth volume (Kofron and Hoffman-Kim, 2009). Laminin is therefore not added because two components are better than one, it is added because it specifically closes the integrin gap that collagen I leaves open in the post-Wallerian axonal membrane.

However, laminin itself leaves a third integrin family unaddressed: the αv and α5 subfamilies (αvβ3, αvβ5, α5β1), which are activated downstream of Nogo-receptor signaling and mediate elongation through the PI3K/mTOR axis. Neither collagen I nor laminin presents the RGD sequence these integrins recognize. Together, αv and α5 integrins account for approximately 80% of RGD-mediated adhesion in integrin-ligand binding studies, while laminin peptides IKVAV and YIGSR engage primarily α3β1, α4β1, and α6β1, confirming the two systems are complementary rather than redundant (Ruoslahti, 1996). Full fibronectin, which natively carries the RGD sequence, is excluded because it was shown to be slightly inhibitory to neurite extension in direct co-gel experiments (Kofron and Hoffman-Kim, 2009). Isolated RGD peptides presented on a neutral carrier deliver the adhesive signal without this inhibitory context, and short RGD sequences have been shown to bind αv and α5 integrins even on partially compromised membrane surfaces, precisely the post-Wallerian condition these axons present (Ruoslahti and Pierschbacher, 1987).

The three-component adhesion system (collagen I, laminin, RGD peptides) therefore emerges from a single deductive chain: collagen I is the injectable fibrillar vehicle; laminin closes the α3/α6/α7 integrin gap left by Wallerian degeneration; and RGD peptides close the αv/α5 gap that laminin cannot address. Together, the three components cover every major integrin family expressed on regenerating axonal membranes and growth cones in the post-injury environment.

##### 2.2.2.3 Deficit in molecular guidance

Clinical question: Can directed, sustained chemotactic gradients be established within the lesion environment to guide regenerating axons toward topographically correct synaptic targets?

Molecular Guidance of axonal growth requires not only the presence of neurotrophic factors but their spatial organization as concentration gradients with a defined vector. The literature distinguishes between non-directional trophic support (general survival and elongation) and genuine guidance [vectorial orientation of the growth cone (Song et al., 1997)]. Both properties are required for functional reconnection. In this context, these principles are considered within the framework of the scaffold, which serves as the structural basis for gradient formation and maintenance. Building on this, the most relevant studies addressing these mechanisms will be comparatively analyzed to identify optimal strategies for their implementation.

**Table:** 

Study	Intervention	Key finding	Relevance
Song et al., 1997	BDNF gradient (*in vitro*)	Growth cones turned toward increasing BDNF concentrations; reversal of gradient reversed turning direction.	Demonstrates BDNF provides vectorial guidance, not just trophic support
Genç et al., 2004	NT-3 deletion/rescue	Proprioceptive axons showed severe topographic errors in NT-3-null animals; NT-3 re-expression restored correct targeting.	Establishes NT-3 as an obligate guidance cue for proprioceptive pathways
Cao and Shoichet, 2003	Combined BDNF + NT-3 gradient	Dual neurotrophin gradients produced synergistic increase in axonal extension and orientation compared to each factor alone.	Directly justifies co-embedding BDNF and NT-3 in Hydrogel II to maximize guidance precision.
Kobayashi et al., 1997	BDNF and NT-4/5 delivered to axotomized rubrospinal neurons in rat cervical SCI	Both neurotrophins prevented neuronal atrophy, upregulated regeneration-associated genes GAP-43 and Tα1-tubulin, and promoted axonal regrowth	Confirms BDNF as a sustaining delivery requirement for axonal regeneration beyond simple trophic support, reinforcing the controlled-release rationale of the TEM
Blesch and Tuszynski, 2007	NT-3 + fibroblast grafts	Sensory axons regenerated into NT-3-secreting grafts and extended beyond them when the gradient was maintained.	Demonstrates that NT-3 gradients must be sustained and directional — informs controlled-release design of TEM.

Across the five studies reviewed, two neurotrophins emerge as the most robustly evidenced chemical guidance agents for spinal cord regeneration: BDNF and NT-3. Song et al. (1997) established that BDNF is not merely trophic but genuinely chemotropic — growth cones reorient in real time according to gradient direction — while Kobayashi et al. (1997) confirmed that this effect requires sustained delivery, ruling out bolus approaches. For proprioceptive and sensory pathways, Genç et al. (2004), Blesch and Tuszynski (2007) converge on NT-3 as an obligate and irreplaceable guidance molecule whose spatial gradient must be maintained continuously; gradient collapse was sufficient to stall or reverse regeneration.

The critical advance provided by Cao and Shoichet (2003) is the demonstration that BDNF and NT-3 act synergistically when co-delivered as spatially organized gradients, producing orientation and extension superior to either factor alone. This synergy is pathway-specific: BDNF preferentially guides motor and corticospinal axons, NT-3 guides proprioceptive and sensory afferents. Spatially offset release peaks within the hydrogel matrix can therefore serve different axonal populations in register with their tract-level topography.

These findings collectively define three non-negotiable design requirements for the chemical guidance subsystem of the TEM. First, both BDNF and NT-3 must be present simultaneously, not sequentially, as their synergistic interaction depends on co-occupancy of the gradient space. Second, delivery must be sustained over the full regeneration window (weeks to months), necessitating controlled-release encapsulation rather than surface adsorption or bolus injection. Third, each factor must form a directional gradient with a defined source-to-target vector; isotropic or homogeneous distributions provide trophic support but abolish chemotropic guidance.

##### 2.2.2.4 Glial scar

Bradbury et al. (2002) established the foundational proof of concept: Chondroitinase ABC (ChABC), a bacterial lyase derived from Proteus vulgaris, degrades the glycosaminoglycan side chains of chondroitin sulfate proteoglycans (CSPGs), the principal biochemical component of the inhibitory glial scar. In their rat SCI model, intrathecal ChABC delivery following dorsal column injury promoted functional recovery of forelimb reaching and gait, with histological evidence of axonal sprouting and serotonergic fiber ingrowth across the lesion site. Critically, efficacy was contingent on a permissive surrounding environment: ChABC alone was insufficient in chronically inflamed tissue, but in conjunction with anti-inflammatory conditioning it consistently enabled regeneration (Bradbury et al., 2002).

The central pharmacokinetic obstacle to clinical ChABC translation is well-characterized and quantitatively severe. Unstabilized ChABC loses 50% of its enzymatic activity within 1 h of incubation at 37 °C, and residual activity is obliterated within 72 h (Raspa et al., 2019). Activity is further diminished within 24 h at 39 °C — the temperature of a febrile post-surgical patient — making the therapeutic window acutely sensitive to systemic inflammatory state (Raspa et al., 2019). At the same time, the pathological environment that ChABC must address persists for weeks to months. CSPG accumulation within the glial scar is not a transient event: reactive astrocytes continuously synthesize brevican, neurocan, versican, and aggrecan during the subacute and chronic phases of SCI (Bradbury and Burnside, 2019), and newly myelinating oligodendrocytes born post-lesion provide additional inhibitory surface molecules. A therapeutic window of 72 h is therefore categorically insufficient for meaningful tissue remodeling.

Literature offers two complementary strategies to overcome this limitation.

Strategy 1: Physical entrapment in the TEM hydrogel matrix — immediate and sustained protein-phase release

Rossi et al. (2013) demonstrated the first clinically viable solution to the ChABC delivery problem: physical entrapment within an agarose–carbomer hydrogel. ChABC molecules were incorporated within the three-dimensional pore network during the sol-gel transition, prior to crosslinking. Because the enzyme is entrapped rather than covalently bound, no chemical modification of the protein occurs and its tertiary structure — and therefore its catalytic activity — is preserved. Release occurs by diffusion through the hydrogel pores over a 1-week period, driven by the concentration gradient between the gel interior and surrounding tissue. The key pharmacokinetic finding was that enzymatic activity was confirmed at days 1, 2, 5, and 7 by SDS-PAGE assay of decorin digestion products, with the 45 kDa decorin core protein diagnostic of complete GAG chain cleavage present at every time point — demonstrating that agarose–carbomer entrapment is biochemically neutral with respect to ChABC’s molecular structure (Rossi et al., 2013).

This 7-day release window directly addresses the most critical phase of the present protocol: the period immediately following scaffold implantation when newly deposited CSPGs from reactive astrocytes are most abundant and the lesion is most susceptible to enzymatic intervention. Early CSPG digestion prevents consolidation of the inhibitory biochemical field within and around the scaffold, maintaining the permissive corridor through which regenerating axons must extend.

The protein-phase window can be extended beyond 7 days through stabilization strategies compatible with the collagen I/RGD/laminin matrix of Hydrogel II. Raspa et al. (2019) showed that sucrose stabilization at 2.5 mol/L — incorporated directly into the hydrogel solution prior to gelation — preserved ChABC enzymatic activity *in vitro* for up to 14 days at 37 °C. Lee et al. (2010) extended this further using trehalose stabilization combined with lipid microtube delivery: thermostabilised ChABC retained full enzymatic activity for up to 4 weeks *in vitro* and maintained *in vivo* CSPG depletion for at least 6 weeks post-SCI.

Strategy 2: Viral vector delivery — sustained *in situ* expression for the elongation and consolidation phases

Protein-phase release, even when stabilized to 4 weeks, cannot cover the full temporal requirement for CSPG management. The active axonal elongation phase extends over weeks to months, and synaptic consolidation and remyelination may persist for longer still. Any resurgence of CSPG accumulation during this period risks re-inhibiting growth cones that have not yet reached their targets (Blesch and Tuszynski, 2007; Bradbury and Burnside, 2019).

Burnside et al. (2018) resolved this gap through gene therapy: an immune-evasive adeno-associated viral (AAV) vector encoding ChABC provided regulated, sustained *in situ* expression of the enzyme within the injured spinal cord for at least 12 weeks following a single injection. The immune-evasive capsid design critically reduces the adaptive immune response that limits repeated dosing of the bacterial protein — particularly important given that repeated systemic or local ChABC administration can trigger antibody-mediated neutralization (Burnside et al., 2018). Lentiviral vectors offer an alternative route with long-term genomic integration, but their insertional mutagenesis risk makes AAV — particularly serotypes AAV5 and AAV9, which efficiently transduce both neurons and glia — the preferred option for this protocol given its established clinical safety record (Burnside et al., 2018). Viral particles can be embedded directly within Hydrogel II prior to injection or administered as a separate intraparenchymal injection 48–72 h after TEM placement.

The viral expression onset typically lags 7–14 days behind injection, corresponding to the time required for AAV transduction, nuclear entry, and transcription/translation. This lag is pharmacokinetically advantageous: viral-phase ChABC production begins to ramp up precisely as protein-phase release from the hydrogel is declining, creating a seamless handover between the two delivery mechanisms with no gap in CSPG coverage (Burnside et al., 2018; Lee et al., 2010; Raspa et al., 2019).

ChABC delivery strategy comparison table

**Table:** 

PK/stability parameter	Bolus ChABC	Hydrogel-entrapped (hydrogel II)	Viral vector (AAV/LV)
Enzymatic t1/2 at 37 °C	<1 h (50% activity lost within 1 h; obliterated within 72 h)	Up to 28 days (trehalose + microtubes)	Continuous (weeks–months via *in situ* expression)
Active CSPG degradation window	<3 days; repeat injections every 48 h	Up to 6 weeks (thermostabilized microtube system)	≥12 weeks (AAV5 single injection)
pH/temp optimum	pH 8.0/37 °C — highly sensitive to deviations	pH 8.0 maintained by hydrogel buffer; thermal protection via stabilizing excipient	Physiological conditions; no direct thermal exposure
Aggregation risk	High at 37 °C when concentration is increased to compensate short t1/2	Low — spatial entrapment prevents bulk aggregation	None — enzyme produced locally at controlled levels
Immunogenic risk	Moderate — repeated bacterial protein injections may trigger adaptive response	Reduced — hydrogel limits systemic exposure; biodegradable materials low immunogenicity	Low with immune-evasive vectors; requires careful AAV serotype selection
Delivery invasiveness	High — requires chronic catheter or minipump for sustained delivery	Low — single intraparenchymal injection at TEM implantation	Low — single vector injection; may require surgical pre-transduction

Summary: ChABC can be incorporated in Hydrogel II via physical entrapment with sucrose or trehalose stabilization (protein phase, days 0–14 to 28). A viral vector adjunct (AAV, days 2–3 post-TEM) provides seamless handover to sustained *in situ* expression (weeks 4–12+).

**Table:** 

	Collagen I	Laminin	RGD peptides	BDNF	NT-3	ChABC
Collagen I	–	Compatible no receptor overlap (Plantman et al., 2008)	Compatible Col-I targets α1/α2 (Ruoslahti and Pierschbacher, 1987; Ruoslahti, 1996)	Compatible BDNF bioactivity preserved (Han et al., 2009)	Compatible NT-3 bioactivity preserved (Han et al., 2009)	Compatible chains only; not collagen (Bradbury et al., 2002)
Laminin	see Col-I/Lam	–	Compatible distinct binding domains (Plantman et al., 2008; Ruoslahti, 1996)	Compatible fully independent cascades (Plantman et al., 2008)	Compatible fully independent cascades (Plantman et al., 2008)	Compatible ChABC leaves it intact (Bradbury et al., 2002; Massey et al., 2008)
RGD peptides	see Col-I/RGD	see Lam/RGD	–	Compatible orthogonal receptor systems (Ruoslahti and Pierschbacher, 1987)	Compatible orthogonal receptor systems (Ruoslahti and Pierschbacher, 1987)	Compatible ChABC cleaves GAGs only (Bradbury et al., 2002; Ruoslahti and Pierschbacher, 1987)
BDNF	see Col-I/BDNF	see Lam/BDNF	see RGD/BDNF	–	Synergistic combined effect (Cao and Shoichet, 2003)	Compatible degrade BDNF (Massey et al., 2008)
NT-3	see Col-I/NT-3	see Lam/NT-3	see RGD/NT-3	see BDNF/NT-3	–	Synergistic sprouting than either alone (Massey et al., 2008)
ChABC	see Col-I/ChABC	see Lam/ChABC	see RGD/ChABC	see BDNF/ChABC	see NT-3/ChABC	–

Clinical question: Is therefore the coformulation of collagen I, laminin, RDG peptides, BDNF, NT-3 and ChABC compatible and synergistic?

The three structural components of Hydrogel II, collagen I, laminin, and RGD peptides, are each biochemically compatible with ChABC co-formulation, and none constitutes a substrate for its enzymatic activity. ChABC is a lyase with strict substrate specificity: it cleaves the glycosaminoglycan side chains of chondroitin sulfate and dermatan sulfate proteoglycans, acting exclusively on the sulfated disaccharide repeats of these chains (Bradbury et al., 2002). Type I collagen is a fibrillar protein with no glycosaminoglycan side chains and no chondroitin sulfate modifications — its triple-helical domain is not a ChABC substrate, and co-entrapment within the same hydrogel matrix does not alter collagen fibrillogenesis or mechanical integrity (Rossi et al., 2013). Laminin is a large glycoprotein that carries heparan sulfate rather than chondroitin sulfate modifications; ChABC has no documented enzymatic activity against heparan sulfate chains, leaving laminin structure and its integrin-binding domains (including IKVAV and YIGSR) fully intact upon co-delivery (Bradbury et al., 2002). RGD peptides are short synthetic tripeptide sequences with no glycosaminoglycan chemistry whatsoever and are therefore entirely outside the catalytic range of ChABC (Ruoslahti and Pierschbacher, 1987). Critically, Massey et al. (2008) demonstrated directly that ChABC retains full enzymatic activity when co-delivered with neurotrophins including BDNF and NT-3 within a hydrogel matrix, confirming that neither the scaffold proteins nor the chemical guidance agents interfere with ChABC catalytic function or vice versa. This mutual biochemical inertness is what permits all five components to be co-formulated in a single injection without requiring sequential or spatially segregated delivery. Below is a summarized schematic table of biomaterial compatibility:

##### 2.2.2.5 Wallerian debris

Clinical question: Once the IL-10-mediated inflammatory suppression of Phase I has slowed Wallerian degeneration and preserved axonal surface integrity long enough for TEM integration, can the resulting debris be efficiently cleared without destructive inflammation and without interfering with the scaffold, guidance, or scar-dissolution components already in place?

The role of Phase I in setting up this barrier and macrophages

The IL-10 component of Hydrogel I plays a dual role. Its primary function is DAMP suppression, already established (Shen et al., 2022). Its secondary role is to slow Wallerian degeneration itself: by suppressing IL-1β and TNF-α, which accelerate axonal NAD+ depletion via the SARM1 cascade (Gerdts et al., 2015), Hydrogel I extends the window during which axonal surface integrins remain available for TEM adhesion — an emergent benefit of the same strategy already selected for inflammation control. That said, Wallerian degeneration must ultimately occur to open the physical corridor for TEM integration. For this, macrophages and resident microglia are key regulators, adopting divergent phenotypes that critically determine regenerative outcome.

M1 vs. M2 phenotype: what the distinction means in practice

M1 macrophages are driven by pro-inflammatory signals and rapidly lose their debris-clearing capacity: excessive myelin lipid uptake causes foam cell formation, blocking continued processing (Kigerl et al., 2009). Their effect on neurons is actively harmful — when applied to DRG neurons, M1-conditioned medium produces disorganized, non-directed growth terminating before 500 μm (Kigerl et al., 2009).

M2 macrophages, induced by IL-4 and IL-13, clear debris efficiently without foam cell accumulation, and their conditioned medium produces unipolar DRG extensions exceeding 1 mm — more than twice the M1 output (Kigerl et al., 2009). Beyond debris clearance, M2-derived signals drive oligodendrocyte precursor differentiation, priming the remyelination phase of the protocol at no additional cytokine cost (Fancy et al., 2009; Miron et al., 2013).

**Table:** 

Study	Model	Key insight
Kigerl et al., 2009	Mouse SCI *in vivo*	M1 dominance sustained for weeks post-SCI; M2 markers appear transiently at days 3–7 then decline. M2 conditioned medium induced axon extensions > 1 mm; M1 medium produced growth < 500 μm — a > 2-fold difference. Natural M2 is insufficient and requires therapeutic prolongation.
Matsui et al., 2022	Rat macrophages + peripheral nerve *in vitro*	IL-4 at 10 ng/mL induced M2 phenotype (high CD206, Arg-1) within 48 h with enhanced myelin debris phagocytosis. M2 secretome included uPA, providing a direct pro-regenerative axonal signal independent of neurotrophin gradients.
Wang et al., 2015	Mouse bone marrow macrophages *in vitro*	Myelin debris re-converted M2-primed macrophages back toward M1. Identifies a pathological positive-feedback loop: poor M1 debris clearance → debris accumulation → M2-to-M1 reversion. M2 polarization is unstable without concurrent debris removal — the two processes must reinforce each other.
Miron et al., 2013	Mouse CNS demyelination model	M2 depletion at remyelination onset delayed myelin formation. M2-derived activin-A identified as effector driving OPC differentiation. IL-4/IL-13 stimulus therefore delivers two sequential outputs: debris clearance and remyelination priming from a single intervention.
Van Broeckhoven et al., 2021	Review: mouse and rat SCI	Prolonged M2 stimulation beyond the debris clearance window risks TGF-β-mediated fibroblast activation and pro-fibrotic ECM remodeling. Therapeutic M2 window should be bounded by debris clearance timeline (∼days 7–21 post-TEM). Establishes the termination criterion.

The temporal asymmetry between the two phenotypes in untreated SCI defines the core problem. M1 is rapidly induced and sustained for weeks to months without intervention. M2 appears transiently at days 3–7 and declines in the absence of therapeutic support (Kigerl et al., 2009). The protocol must therefore do two things simultaneously: maintain M2 long enough to complete debris clearance and terminate the stimulus before sustained M2 stimulation triggers TGF-beta-mediated fibroblast activation and pro-fibrotic ECM remodeling, a risk documented by Van Broeckhoven et al. (2021) when M2 polarization extends beyond its functional window (Van Broeckhoven et al., 2021). This temporal constraint is what directly motivates the biodegradable Hydrogel III delivery design rather than a continuous pump or repeated injection approach.

M2 macrophage polarization — evidence base and protocol rationale

Conclusion: M2 polarization via IL-4/IL-13 uniquely enables efficient Wallerian debris clearance, supports axonal growth, and promotes remyelination. However, its therapeutic window is narrow: it must persist beyond the transient 3–7 day endogenous response to ensure clearance yet stop before fibrosis is induced. Importantly, its stability requires prior TLR attenuation by Hydrogel I, making the sequential application of both hydrogels essential.

### 2.3 Synthesis: toward a unified sequential hypothesis

The preceding. Immunomodulation analysis shows that each of the five principal barriers to CNS regeneration can be addressed with evidence-based strategies. Crucially, these interventions are not simply additive but sequentially interdependentmust precede scaffold delivery, structural alignment must precede hydrogel application, and chemoattractant gradients with CSPG degradation must be maintained throughout axonal elongation. Notably, even within this sequential framework, several interventions act synergistically. The integration table below formalizes these dependencies:


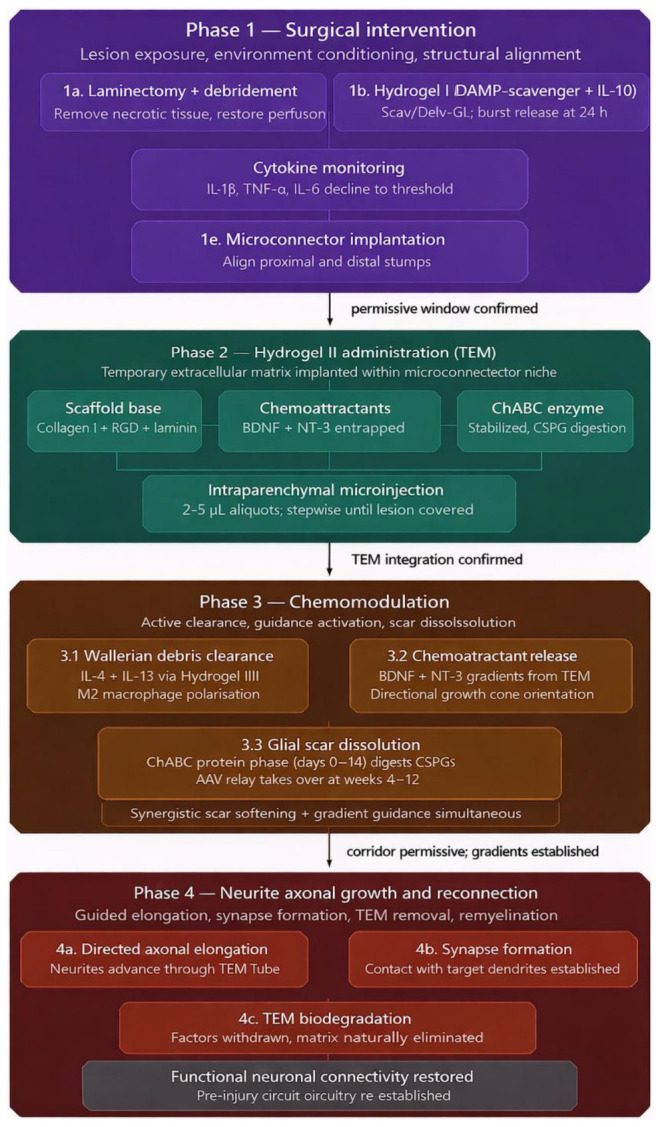


## 3 Hypothesis

We hypothesize that the failure of spinal cord regeneration after acute injury is not attributable to the absence of effective individual therapies, but to the absence of their correct temporal integration. Each of the five principal barriers to CNS axonal regrowth (generalized inflammation, loss of structural guidance, deficit in molecular guidance, glial scar formation, and Wallerian debris persistence) is addressable by existing evidence-based interventions. However, these barriers are causally interdependent: each amplifies the others, and this interdependence imposes a biologically determined sequence of intervention that cannot be violated without compromising the efficacy of subsequent phases. The order of treatment is therefore not a clinical preference but an emergent property of the pathological system itself.

### 3.1 Central hypothesis

multimodal intervention in which the sequence of The central hypothesis of this study is that successful axonal regeneration after acute spinal cord injury requires a temporally ordered, therapeutic phases is dictated by the causal dependencies between the biological barriers being addressed. Specifically, we propose that: early localized immunomodulation must precede scaffold implantation, because unresolved DAMP-driven inflammation prevents TEM integration and reduces axonal surface integrin availability; mechanical realignment must be established before matrix delivery, because scaffold continuity across the lesion depends on physical approximation of the severed stumps; and sustained molecular guidance and CSPG degradation must operate concurrently throughout axonal elongation, because neither alone is sufficient to produce directed reconnection. Disruption of this sequence at any phase is predicted to produce a defined failure mode (impaired scaffold integration, misdirected axonal growth, or scar-mediated stalling) that is distinct from the failure produced by disruption at any other phase. This falsifiable prediction distinguishes the present framework from additive multimodal approaches and provides the basis for a structured preclinical validation design in which each sequential dependency can be tested independently before integration.

## 4 Proposed mechanism

Based on the proposed hypothesis, the literature review, and existing clinical evidence, we present a conceptual, stepwise framework that integrates current knowledge and addresses the central question of this hypothetical study:

### 4.1 Surgical intervention in lesional focus

The objective of this phase is to establish a microenvironment that supports axonal regeneration by first reducing the acute inflammatory response and then restoring structural continuity between severed spinal cord ends via a microconnector.

The sequence of this intervention is structured as follows:

(a) Lesion exposure and structural preparation

After systemic stabilization, the lesion site is surgically exposed via laminectomy. Gentle microsurgical debridement is performed to remove non-viable tissue fragments, clots, and compressive elements while preserving intact white matter tracts. This step reduces mechanical compression, restores perfusion, and limits further release of damage-associated molecular patterns (DAMPs), which are major drivers of secondary injury cascades (Rotshenker, 2011). DAMPs released from injured cells activate innate immune signaling pathways in microglia, astrocytes, and infiltrating macrophages, promoting cytokine release, oxidative stress, and formation of inhibitory glial scars that impede axonal elongation (Bradbury and Burnside, 2019; Van Broeckhoven et al., 2021). Early structural decompression combined with removal of gross necrotic debris has been shown to reduce peak DAMP concentrations at the lesion core and improve tissue perfusion in experimental SCI models (Van Broeckhoven et al., 2021), establishing it as the mandatory first stage of microenvironment conditioning before any pharmacological intervention.

(b) Localized damp-scavenging and immunomodulation

Following structural preparation, a DAMP-binding immunomodulatory hydrogel is applied locally to the lesion site. Shen et al. (2022) showed that a DAMP-scavenging, IL-10–releasing hydrogel can suppress inflammatory signaling and promote regenerative immune responses after spinal cord injury. The composition of the hydrogel is made by a photocrosslinked gelatin with the cationic DAMP-binding polymer poly(amidoamine) and IL-10. Unlike mechanical aspiration approaches, which cannot selectively remove soluble inflammatory mediators, this biomaterial strategy reduces both soluble and diffusible DAMP signaling through biochemical sequestration and controlled cytokine modulation (Shen et al., 2022).

The hydrogel acts through two synergistic mechanisms:

(1) Selective sequestration of extracellular danger signals

Such as nucleic acids, ATP derivatives, and nuclear proteins that perpetuate inflammatory activation (Rotshenker, 2011).

(2) Localized release of anti-inflammatory cytokines (e.g., IL-10)

To promote polarization of macrophages and microglia toward reparative phenotypes (Bethea et al., 1999; Kigerl et al., 2009). The persistence of DAMPs in the spinal microenvironment is a major barrier to regeneration because these molecules maintain inflammatory activation, stimulate astroglial reactivity, and inhibit axonal growth (Rotshenker, 2011; Yiu and He, 2006). By contrast, localized scavenging combined with controlled immunomodulation attenuates this cascade (Shen et al., 2022). Importantly, this phase is designed to be transient rather than continuous. The hydrogel is engineered for self-limited activity through gradual biodegradation, allowing early suppression of excessive inflammation while preserving later physiological repair signaling. Sustained immunosuppression beyond the acute inflammatory phase could impair debris clearance, angiogenesis, and endogenous regenerative pathways (David and Lacroix, 2003; Fancy et al., 2009).

Therefore, immediately after lesion-site preparation, Hydrogel I [IL-10 + polymer poly(amidoamine)] will be locally applied to the injury cavity as a transient biochemical conditioning system designed to neutralize DAMPs and induce controlled anti-inflammatory polarization before any regenerative scaffold implantation. It will be administered using a combined topical and intraparenchymal microinjection approach to ensure uniform coverage of the lesion interface and surrounding inflammatory zones (Shen et al., 2022).

This step establishes a stabilized microenvironment permissive for regeneration while preserving later physiological repair mechanisms, thereby defining the mandatory first phase of the therapeutic sequence. Therapeutic activity will be maintained until inflammatory markers (IL-1 β, TNF-α, IL-6, etc.) decrease to a predefined low-activity threshold, ensuring sufficient suppression of the hostile microenvironment. Once this state is achieved, administration is discontinued to avoid excessive immunosuppression and to permit physiological repair processes (Fancy et al., 2009).

In addition to stabilizing the inflammatory microenvironment, this transient immunomodulatory phase is expected to slow the progression of Wallerian degeneration, thereby preserving axonal architecture and molecular surface cues during the critical early post-injury window. By attenuating cytokine-driven proteolysis, oxidative stress, and secondary cytoskeletal breakdown, early suppression of inflammatory signaling prolongs the structural integrity of peri-axonal substrates that would otherwise be rapidly degraded (Gerdts et al., 2015).

Pharmacokinetics of hydrogel I: The release kinetics and degradation profile of this hydrogel system are critical and interdependent design parameters that must be considered jointly when translating the scaffold to human application. As characterized by Shen et al. (2022), the IL-10 release follows a biphasic exponential profile: an initial burst of approximately 24% of the total loaded cytokine is released within the first 24 h, after which release slows to a sustained gradient that leaves approximately 52.4% of the IL-10 still retained within the matrix at day 21. Concurrently, the scaffold itself undergoes gradual biodegradation, losing approximately 25% of its dry mass by day 28 *in vitro*, and reaching complete elimination by 8 weeks post-implantation *in vivo*, a timeline confirmed by H&E histology in the murine SCI model. These two curves are not independent: as the gelatin matrix degrades, crosslink density decreases, pore size increases, and diffusional resistance drops, which progressively accelerates IL-10 release during the later sustained phase. The scaffold therefore functions as a self-dissolving delivery system in which the degradation curve directly modulates the shape.

**Figure:**
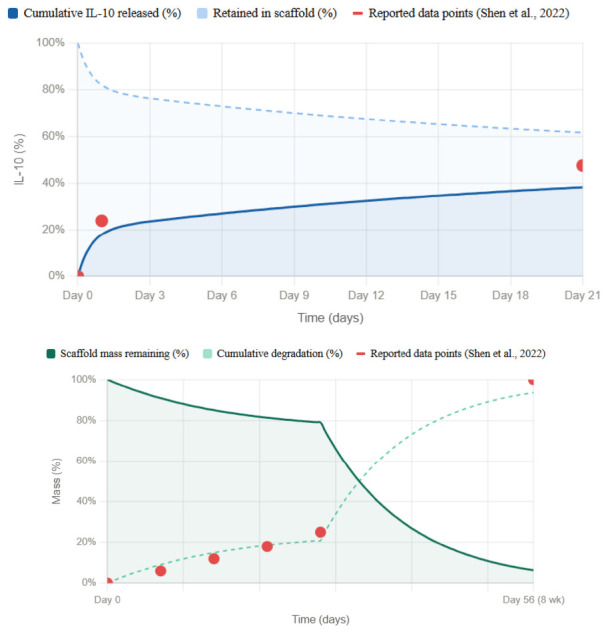
Release curve of IL-10 in Hydrogel I. illustrative mechanistic estimates based on data reported by Shen et al. (2022). Degradation curve of the DAMP-scavenging-scaffold. Illustrative mechanistic estimates, based on data reported by Shen et al. (2022).

In translating this system to human application, the substantially larger lesion volume, slower immune dynamics, and prolonged secondary injury window, which may span weeks rather than days, will require recalibration of all three compositional variables simultaneously. Gelatin concentration governs crosslink density and therefore controls both the degradation rate and the diffusional resistance to IL-10 release; increasing it will flatten and extend both curves. PAMAM-G3 content modulates electrostatic retention of IL-10 within the matrix, effectively delaying the burst phase and reducing the initial release fraction while extending the sustained phase. IL-10 loading dose sets the total cytokine reservoir and scales the absolute amount delivered at each timepoint without altering the curve shape. By adjusting these three parameters in concert, both the release curve and the degradation curve can be co-tuned — shifting the burst window, extending the sustained plateau, and aligning scaffold persistence with the broader inflammatory timeline of human spinal cord injury.

(c) Temporary pharmacological stabilization (optional adjunct)

A short-acting corticosteroid such as dexamethasone may be administered during the hyperacute phase as an adjunctive stabilization measure. Early glucocorticoid treatment in preclinical spinal cord injury models has been shown to reduce microglial activation, pro-inflammatory cytokine release, edema, and tissue loss, resulting in improved preservation of neural structures and functional outcomes (Bracken et al., 1997). However, because corticosteroids broadly suppress immune activity, their use is restricted to a brief early window and is not intended as a sustained therapy (Bracken et al., 1997). Their role is supportive and transitional, bridging the interval until local hydrogel-mediated immunomodulation establishes a stable microenvironment (Shen et al., 2022).

Similarly, early administration of IL-10 has demonstrated neuroprotective effects in experimental spinal cord injury. Acute delivery shortly after trauma reduces TNF-α levels, decreases lesion volume, and improves motor recovery scores, indicating that transient cytokine modulation can attenuate secondary inflammatory injury (Bethea et al., 1999). In the present protocol, IL-10 is preferentially delivered locally through hydrogel rather than via repeated systemic administration, allowing spatially restricted and temporally controlled exposure (Shen et al., 2022).

(d) Microenvironment conditioning interval

After hydrogel application, a short conditioning interval is allowed to enable reduction of inflammatory mediators, stabilization of the lesion environment, and initiation of reparative immune polarization (Kigerl et al., 2009; Shen et al., 2022). This interval is essential for minimizing oxidative stress, proteolytic activity, and cytokine toxicity before implantation of structural regenerative devices (David and Lacroix, 2003; Gerdts et al., 2015; Rotshenker, 2011).

(e) Microconnector implantation

In complete spinal cord transection, a frequent problem is the loss of physical continuity between the proximal and distal stumps. Once severed, both ends retract and become spatially disconnected, eliminating any intrinsic guidance for axonal regrowth (David and Lacroix, 2003; Thuret et al., 2006). As a result, regenerating axons lack a defined trajectory and cannot bridge the lesion in an organized or functional manner (Thuret et al., 2006; Yiu and He, 2006). Therefore, before any biochemical or scaffold-based strategy can be effective, a macroscopic guiding structure that restores the original spinal axis and physically reconnects both ends is required (Brazda et al., 2013).

Brazda et al. (2013) described the design and function of the mechanical microconnector, demonstrating that its microchannel architecture and controlled low-pressure application allow stable approximation of severed spinal cord ends while generating a protected internal niche for subsequent interventions. Later, Brazda et al. (2016) published a detailed murine implantation protocol, showing that combining microsurgical exposure, meticulous lesion-site preparation, and precise device placement can consistently reproduce structural tissue alignment in a technically reproducible manner. Finally, Brazda et al. (2016) provided functional evidence that mechanical alignment via the microconnector promotes formation of a neurovascular tissue bridge, facilitates axonal regeneration through the device, and enables localized delivery of therapeutic agents within a controlled microenvironment.

Together, these studies support the plausibility that lesion-site conditioning combined with mechanical micro-alignment can optimize regenerative conditions in complete spinal cord injury (Brazda et al., 2013, 2016; Matsui et al., 2022).

**FIGURE 6:**
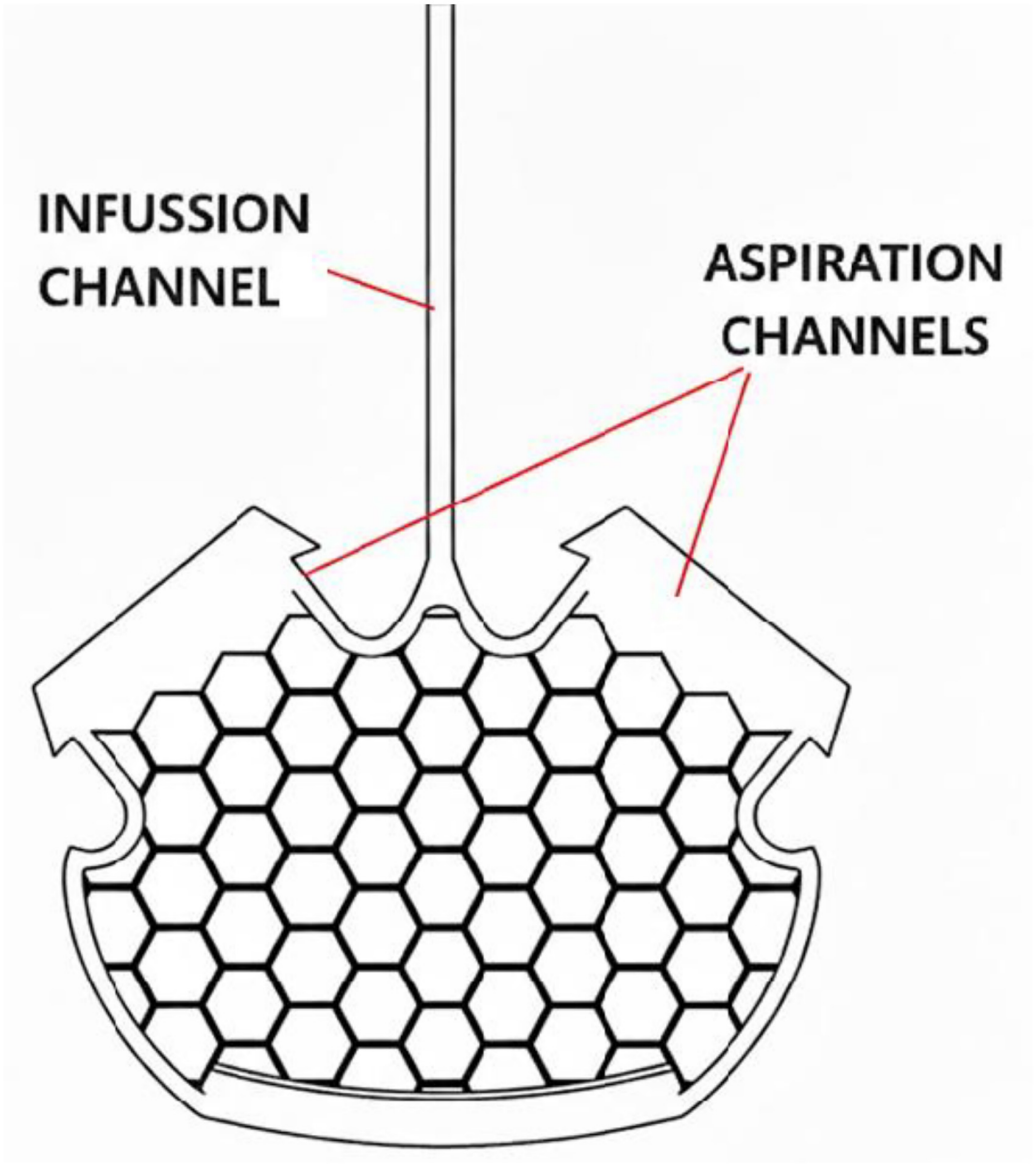
Basic design of the microconnector. Original image based on the design of Brazda et al. (50) “A mechanical microconnector system for restoration of tissue continuity and long-term drug application into the injured spinal cord.”

Once the lesion environment has been stabilized, the microconnector is implanted to mechanically align proximal and distal spinal cord stumps and create a protected internal niche supporting axonal growth and future TEM implantation (Brazda et al., 2013, 2016). Under microscopic guidance, the stumps are carefully inserted into the device channels and oriented parallel to the spinal axis to ensure optimal structural continuity (Brazda et al., 2016). Mechanical alignment at this stage maximizes scaffold integration, enhances survival of residual axons, and provides a controlled microenvironment for subsequent regenerative interventions (Brazda et al., 2016). Here in Figure 6 it is shown a basic frontal view of the microconnector.

### 4.2 Hydrogel II matrix preparation and administration

Following laminectomy, inflammatory stabilization, and microconnector placement, Hydrogel II is prepared and administered to form the temporary extracellular matrix (TEM). This formulation contains type I collagen, laminin, and RGD peptides — a three-component system whose rationale was established in the material selection framework: collagen provides the injectable fibrillar vehicle, laminin closes the α3/α6/α7 integrin gap opened by Wallerian degeneration, and RGD peptides address the residual αv/α5 integrin family that neither collagen nor laminin engages (Dubey et al., 1999; Plantman et al., 2008; Ruoslahti and Pierschbacher, 1987).

Type I collagen forms the structural base of the TEM. Its fibrillar architecture at the scale of growth cone filopodia provides contact guidance for elongating neurites, and its mechanical stiffness is tunable by concentration — gels at lower concentrations of 0.6–0.8 mg/mL produce maximum neurite extension while stiffness increases from 2.2 Pa to 17.0 Pa across the tested range, establishing an inverse relationship between scaffold stiffness and axonal outgrowth that defines the optimal preparation range for this protocol (Willits and Skornia, 2004). Collagen also functions as the delivery vehicle for the other bioactive components, releasing them through diffusion as the matrix progressively degrades (Han et al., 2009).

Laminin is incorporated to expand the adhesion repertoire of the TEM onto post-Wallerian axonal membranes. Its α6β1, α7β1, and α3β1 integrin interactions are distinct from those mediated by collagen, and its bioactive peptide sequences IKVAV and YIGSR provide additional signals for axonal guidance and neuroprotection (Plantman et al., 2008). Quantitatively, collagen–laminin co-gels produce an 85.9% increase in neurite outgrowth volume over collagen-only controls, confirming that laminin’s contribution is functional rather than additive (Kofron and Hoffman-Kim, 2009).

RGD peptides complete the adhesion system by engaging the αv and α5 integrin subfamilies — receptors activated downstream of Nogo signaling and growth cone mechanosensing — which account for approximately 80% of RGD-mediated adhesion in integrin-ligand binding studies (Ruoslahti, 1996).

The three components do not compete for the same receptors and act on complementary integrin families across the axonal membrane, covering the full spectrum of adhesion sites available in the post-injury environment.

#### 4.2.1 Chemoattractants incorporation

**FIGURE 7:**
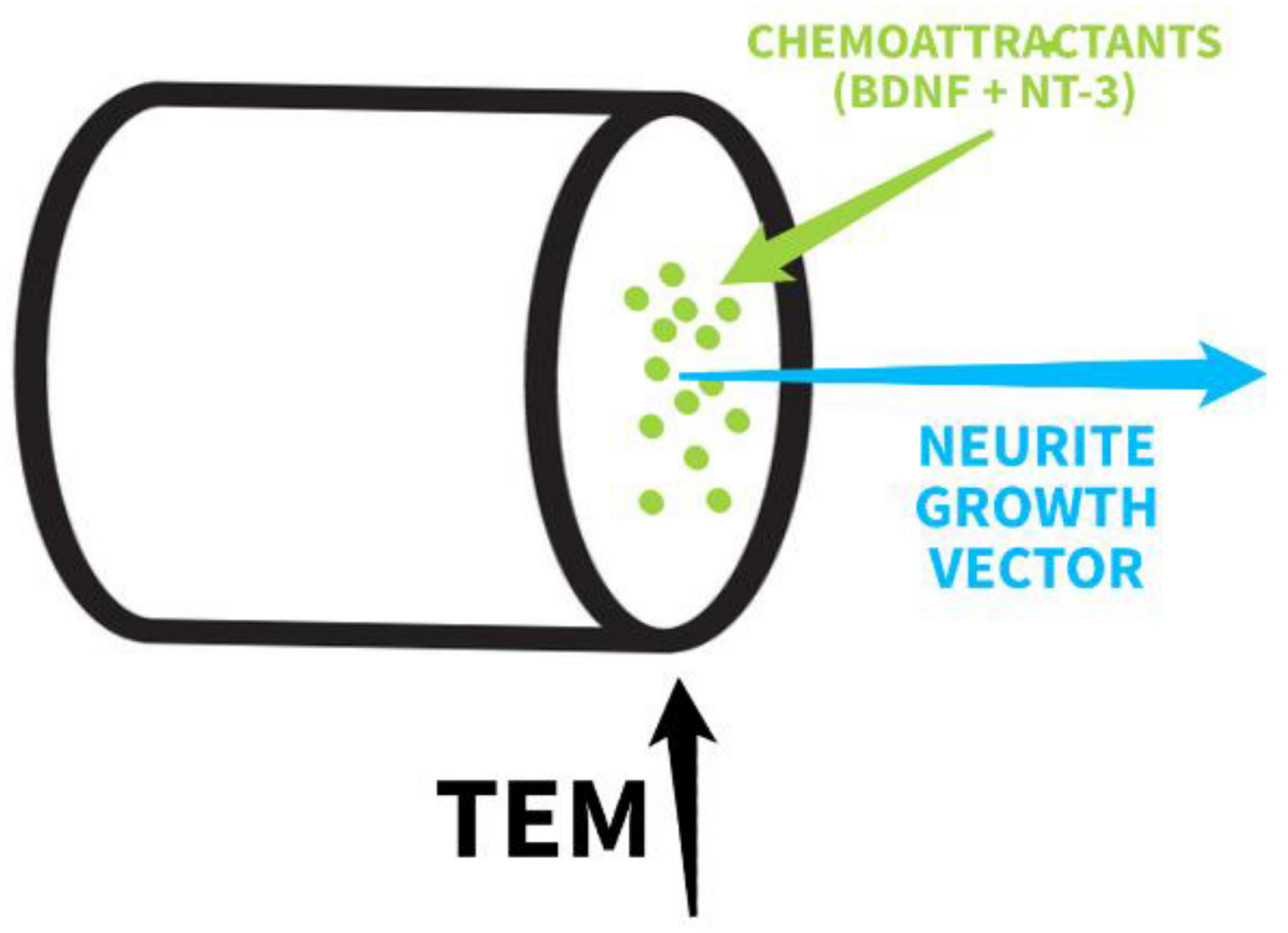
Scheme of chemoattractants and TEM. Original image.

Complementary to the main components of the TEM, the solution of Hydrogel II should incorporate two embedded chemoattractants, designed to provide precise guidance cues once the TEM is established. As the hydrogel crosslinks and forms the scaffold, BDNF and NT-3 become physically entrapped, allowing for gradual and localized release into the central space of the TEM (Han et al., 2009). This controlled release establishes stable chemotactic gradients along the scaffold fibers, ensuring that regenerating neurites are both structurally and chemically guided within the permissive microenvironment (Cao and Shoichet, 2003) as shown in Figure 7. BDNF has been shown to guide growth cones toward increasing gradients in classical Xenopus models (Song et al., 1997), providing directional cues that promote oriented elongation. NT-3, in turn, functions as a critical guidance cue for proprioceptive axons, with clear orientation defects observed in its absence (Genç et al., 2004). Cao and Shoichet (2003) demonstrated that the combined application of BDNF and NT-3 produces synergistic effects, enhancing both neurite extension and guidance precision. By embedding these neurotrophins in Hydrogel II, the TEM not only provides structural support but also serves as a coordinated biochemical highway, aligning physical architecture with trophic signaling to maximize directed axonal regeneration (Blesch and Tuszynski, 2007; Cao and Shoichet, 2003; Han et al., 2009).

#### 4.2.2 ABC chondroitinase incorporation

**FIGURE 8:**
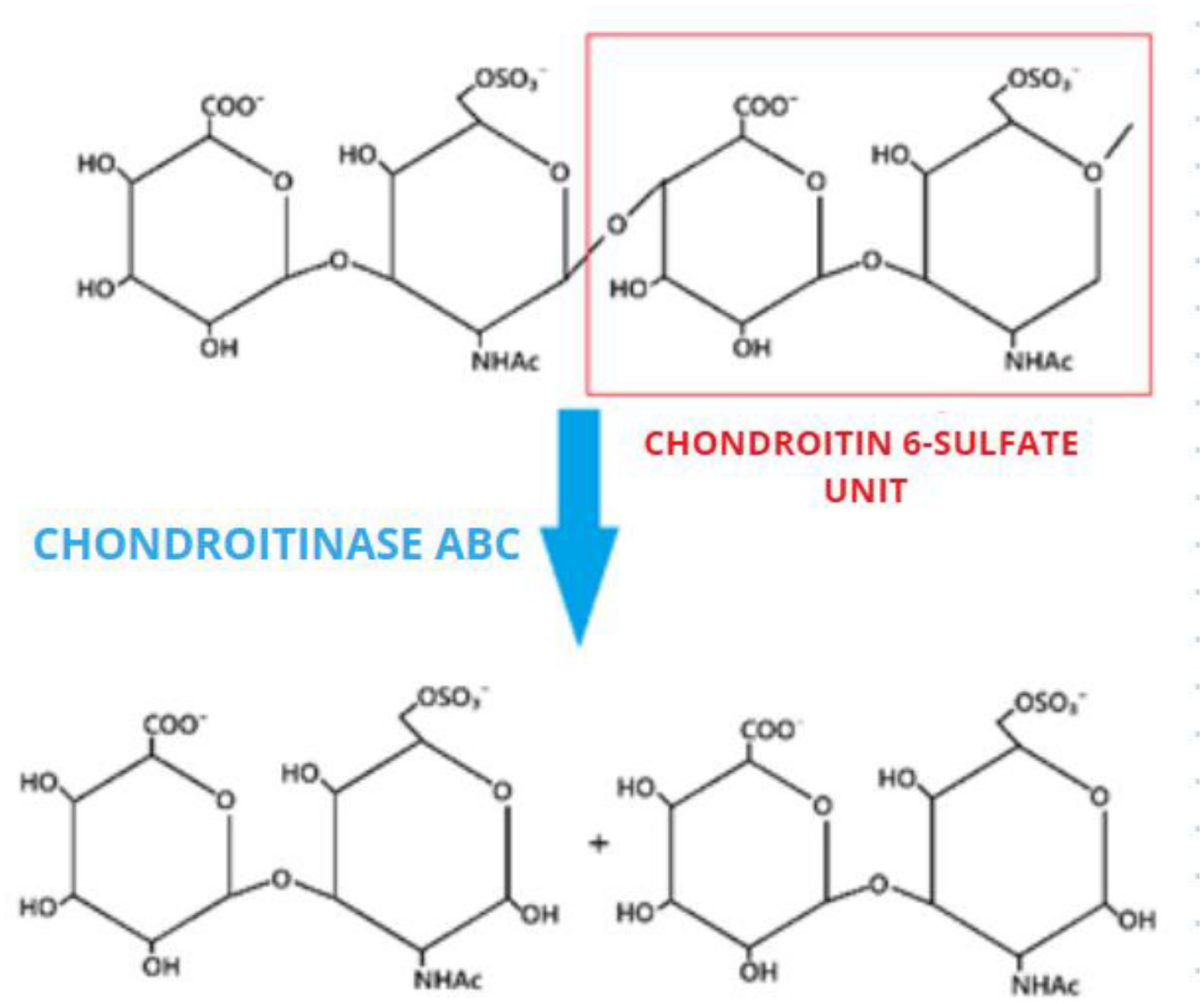
Schematic of the ABC Chondroitinase reaction. Original image inspired by the book Comprehensive Glycoscience (2007) K. Takagaki, I. Kakizaki.

In addition, Bradbury et al. (2002) demonstrated the efficacy of Chondroitinase ABC (ChABC), an enzyme capable of degrading CSPGs, which, when combined with a permissive anti-inflammatory and neuroplastic environment, facilitates the removal of inhibitory components of the glial scar and exposes pre-lesion axonal segments, enabling regeneration. Figure 8 shows a basic scheme on the molecular reaction of ChABC on CSPGs. Complementary to these findings, Rossi et al. (2013) showed that ChABC can be physically entrapped in an agarose–carbomer hydrogel, preserving enzymatic activity while providing sustained, localized release over several days, addressing its inherent thermolability and enzymatic instability. To further extend the protein-phase window, trehalose stabilization has been shown to preserve full enzymatic activity for up to 4 weeks *in vitro*, with *in vivo* CSPG depletion maintained for at least 6 weeks post-SCI (Lee et al., 2010).

This configuration ensures controlled, spatially targeted CSPG degradation along the neurite pathways. To extend enzymatic activity further, viral vector delivery can be employed, either via viral particles embedded in the hydrogel or through pre-transduced supportive cells within the TEM, providing sustained, *in situ* ChABC expression that complements the initial protein release and maintains a permissive extracellular environment over longer periods (Burnside et al., 2018). By combining protein entrapment and viral-mediated expression, the TEM achieves both immediate and prolonged CSPG degradation, effectively softening the lesion and promoting directed axonal growth along the neurotrophin-enriched scaffold (Bradbury et al., 2002; Burnside et al., 2018; Lee et al., 2010)

Therefore, once inflammatory markers have subsided to regenerative-permissive levels and structural alignment has been established through microconnector placement, Hydrogel II is administered via targeted intraparenchymal microinjection to form the TEM (Matsui et al., 2022; Shen et al., 2022). Delivery occurs during the stabilized post-inflammatory window to maximize adhesion to preserved axonal surfaces and residual molecular substrates, ensuring optimal scaffold integration (Plantman et al., 2008; Ruoslahti and Pierschbacher, 1987). Administration is performed stepwise until sufficient matrix coverage and structural continuity across the lesion interface are achieved, as verified by imaging or predefined volumetric parameters (Brazda et al., 2016), after which further delivery ishalted to prevent excessive matrix accumulation or mechanical interference (Zweckberger et al., 2016)

### 4.3 Preparation of hydrogel II

Hydrogel II is prepared as a single injectable formulation combining all five components — type I collagen, laminin, RGD peptides, BDNF, NT-3, and ChABC — in a defined sequence prior to administration. The preparation order is not procedural convention but a biological requirement: all bioactive agents must be incorporated into the collagen solution before fibrillogenesis occurs, so that they are distributed volumetrically throughout the fibrillar network rather than concentrated at the gel surface (Song et al., 1997).

At 4 °C, RGD peptides are first coupled to collagen I monomers in solution, exploiting the temperature-dependent inhibition of collagen self-assembly to ensure homogeneous fibril-surface distribution of adhesion sequences (Song et al., 1997). Laminin is then added to the collagen-RGD solution and mixed homogeneously; pre-gelation mixing is required for laminin to interpenetrate the developing fibrillar network during fibrillogenesis rather than remaining restricted to the gel exterior (Genç et al., 2004). BDNF and NT-3 are subsequently added at concentrations calibrated to establish sustained chemotactic gradients upon scaffold crosslinking (Cao and Shoichet, 2003). ChABC is incorporated last, stabilized with trehalose to preserve enzymatic activity at 37 °C and extend the protein-phase release window up to 4 weeks (Lee et al., 2010). The complete five-component solution is loaded into the delivery system at 4 °C and administered via targeted intraparenchymal microinjection, where physiological temperature drives *in situ* gelation and scaffold conformation to the lesion cavity geometry.

It should be noted that the precise concentrations of each component required for human application have not yet been experimentally determined. The collagen concentration range of 0.6–0.8 mg/mL is established as the optimal window for maximum neurite outgrowth *in vitro* (Willits and Skornia, 2004), and the ChABC stabilization parameters are drawn from published thermostabilizing studies (Lee et al., 2010; Raspa et al., 2019). Laminin loading, RGD peptide density, and neurotrophins concentrations will require iterative *in vitro* and *in vivo* calibration prior to clinical translation, representing a defined experimental gap in the present hypothetical framework.

#### 4.3.1 Pharmacokinetics of hydrogel II


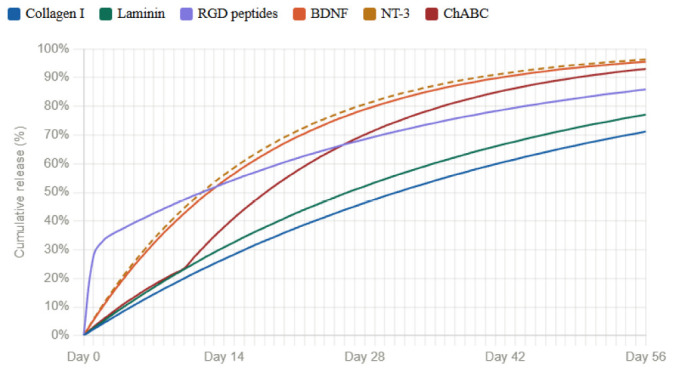


Modeled cumulative release curves based on published mechanistic data. Collagen I: MMP-mediated degradation over 4–8 weeks *in vivo* (Yoshii et al., 2003). Laminin: degradation-coupled diffusion, high molecular weight (∼800 kDa), tracks collagen remodeling timeline (Genç et al., 2004). RGD peptides: biphasic — early surface dissociation burst (∼30% day 1) followed by fibril-coupled sustained release (Ruoslahti and Pierschbacher, 1987). BDNF and NT-3: physically entrapped neurotrophin diffusion over 2–3 weeks (Blesch and Tuszynski, 2007). ChABC: trehalose-stabilized protein phase up to 4 weeks; viral vector (AAV) phase onset day 7–14, sustained ≥ 12 weeks (Burnside et al., 2018; Lee et al., 2010; Raspa et al., 2019). No numerical anchors available for Hydrogel II components; curves represent mechanistic estimates only.

In relation to the degradation curve, the five components of Hydrogel II follow a hierarchical degradation sequence that is mechanistically linked through the collagen fibrillar network and is functionally aligned with the biological timeline of axonal regeneration. RGD peptides are the first to deplete: their small molecular size and surface-coupled distribution result in rapid early dissociation from fibril surfaces within the first 1–3 days, providing a front-loaded integrin adhesion signal precisely during the critical window of initial growth cone contact with the scaffold (Ruoslahti and Pierschbacher, 1987). BDNF and NT-3 follow a moderate depletion curve over 2–3 weeks, releasing by diffusion through the hydrated fibrillar network as pore size increases with early matrix remodeling — a timeline consistent with the active axonal elongation phase that neurotrophin gradients must support (Blesch and Tuszynski, 2007). Laminin, by virtue of its high molecular weight (∼800 kDa), is retained within the fibrillar network substantially longer, with its depletion rate closely coupled to collagen fibril degradation by matrix metalloproteases rather than free diffusion (Genç et al., 2004). The collagen scaffold itself undergoes MMP-mediated degradation over 4–8 weeks *in vivo*, as confirmed in collagen implant studies in spinal cord injury models (Yoshii et al., 2003), defining the outer structural boundary of TEM function. ChABC protein-phase activity declines progressively after day 14–21 despite trehalose stabilization (Lee et al., 2010), but this depletion is compensated by the viral AAV expression phase which begins ramping up at approximately day 7–14 post-injection and sustains *in situ* ChABC production for at least 12 weeks (Burnside et al., 2018). Critically, because all component depletion rates are governed by or coupled to the master collagen degradation clock, adjusting collagen concentration during preparation shifts all timelines simultaneously — a constraint that must be accounted for when calibrating the formulation for human lesion dimensions

### 4.4 Hydrogel III administration

Once the regenerative matrix is adhered to the axonal surface, the next objective is to support neurite outgrowth, which faces three main challenges. First, complete removal of Wallerian debris within the TEM is essential, as remnants of the degenerated post-lesion axon hinder axonal extension (Kigerl et al., 2009; Rotshenker, 2011). Second, following debris clearance, axonal growth requires firm guidance cues to establish correct trajectories, necessitating precise coordination of chemoattractants (Cao and Shoichet, 2003; Song et al., 1997). Third, te hinhibitory glial scar must be addressed, as it represents a physical and biochemical barrier to axonal elongation (Bradbury and Burnside, 2019; Bradbury et al., 2002). This section addresses these three challenges sequentially:

#### 4.4.1 Wallerian remains elimination

Once the TEM is established, the neural environment is primed for the orderly and definitive removal of Wallerian remnants. This phase represents a deliberate shift in strategy: whereas the preceding stages prioritized retention of post-injury axonal architecture to preserve integrin binding sites and molecular surface information for TEM adhesion (Plantman et al., 2008; Ruoslahti and Pierschbacher, 1987), the present phase prioritizes active clearance of that same debris now that scaffold integration is complete.

Retaining Wallerian remnants beyond this point becomes counterproductive — myelin-derived inhibitors and disorganized axonal fragments physically obstruct growth cone progression and, critically, can re-convert M2-polarized macrophages back toward a destructive M1 phenotype through TLR2/4 activation, creating a pathological positive-feedback loop that must be interrupted (Kigerl et al., 2009).

**FIGURE 9:**
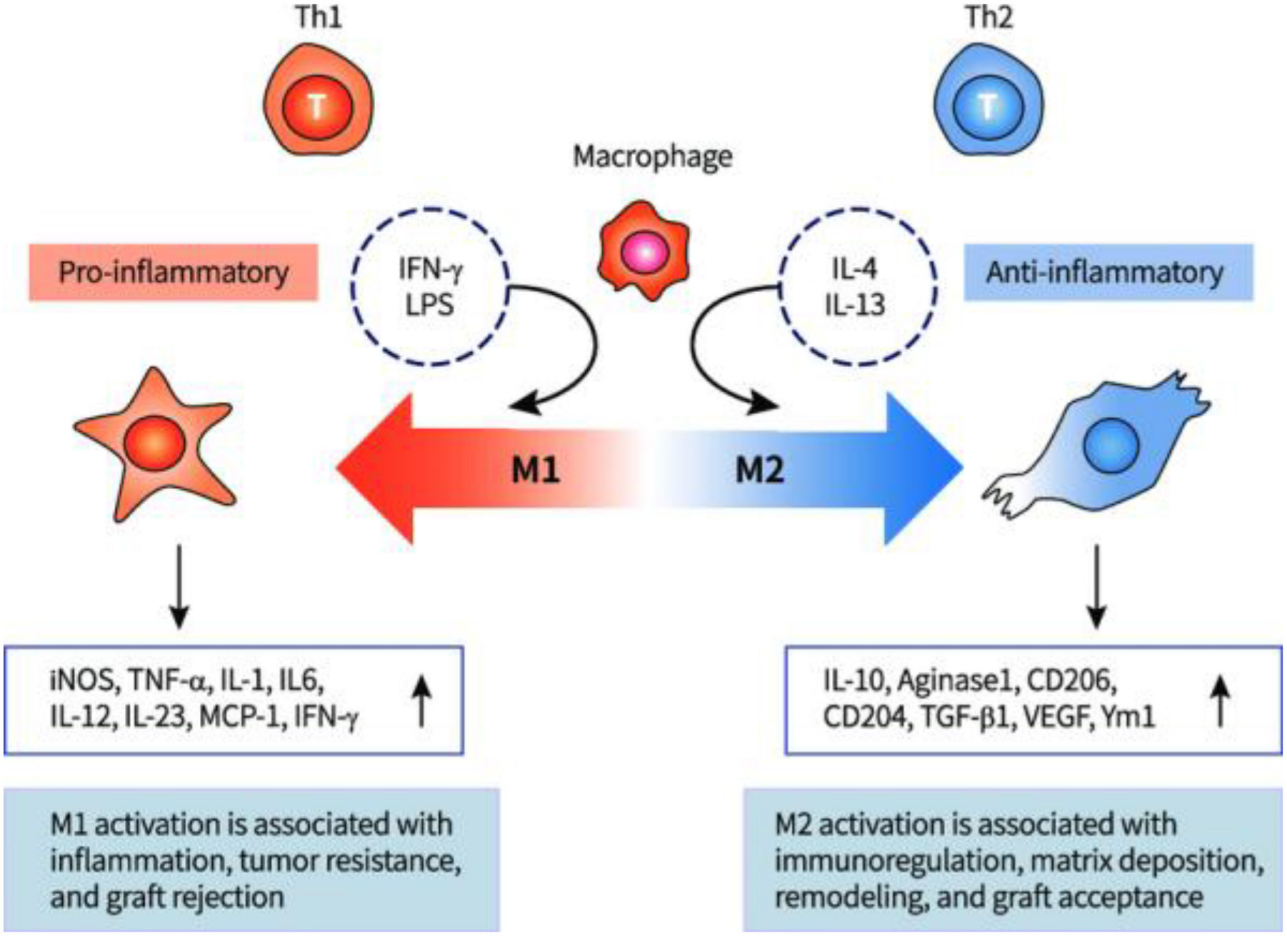
Schematic of the polarization pathways toward the M1 (pro-inflammatory) and M2 (anti inflammatory and reparative) phenotypes. Adapted from [14b] under the terms of the Creative Commons Attribution License. Copyright 2019, Medical Biological Science and Engineering.

The optimal clearance strategy is promotion of macrophage polarization toward the M2 phenotype via IL-4 and IL-13 as shown in Figure 9. As established in the theoretical framework, M1 macrophages stall rapidly upon myelin lipid uptake through foam cell formation and produce a conditioned medium that limits DRG neurite extension to under 500 μm, whereas M2 macrophages clear debris efficiently through TREM2-mediated endocytosis without foam cell accumulation and produce a secretome that supports unipolar extensions exceeding 1 mm (Kigerl et al., 2009). Matsui et al. (2022) confirmed that IL-4 at 10 ng/mL induces a strongly pro-regenerative M2 phenotype within 48 h, with enhanced phagocytic capacity and secretion of uPA — a direct pro-regenerative axonal signal orthogonal to the neurotrophin gradients already established within the TEM. IL-13 provides a complementary M2 induction signal through the same STAT6 pathway, broadening the cytokine stimulus and reducing the risk of incomplete polarization (Kigerl et al., 2009). Beyond debris clearance, M2-derived activin-A drives oligodendrocyte precursor differentiation, priming the remyelination phase at no additional cytokine cost (Fancy et al., 2009).

However, M2 polarization is only stable once myelin debris load is declining — sustained debris exposure re-activates TLR signaling and drives phenotypic reversion regardless of cytokine stimulus (Kigerl et al., 2009). This sequencing dependency is what makes Phase I mandatory before Phase III: Hydrogel I must have attenuated TLR sensitization sufficiently for IL-4/IL-13 to establish and maintain stable M2 polarization rather than being continuously overridden by debris-driven M1 signals.

#### 4.4.2 HYDROGEL III pharmacokinetics and administration

IL-4 and IL-13 will be delivered via a biodegradable Hydrogel III administered once TEM integration is confirmed. An initial higher-dose phase induces rapid M2 polarization and supports peak debris clearance during days 7–21 post-TEM implantation (Fancy et al., 2009; Kigerl et al., 2009). Once the majority of Wallerian remnants have been removed and inflammatory markers have declined to baseline, cytokine delivery is tapered and Hydrogel III undergoes complete biodegradation, terminating the M2 stimulus before sustained IL-4/IL-13 signaling triggers TGF-β-mediated fibroblast activation and pro-fibrotic ECM remodeling (Van Broeckhoven et al., 2021). This time-limited design is not a precaution — it is a biological necessity dictated by the dual role of M2 macrophages as reparative agents within their functional window and fibrogenic drivers beyond it

**Figure:**
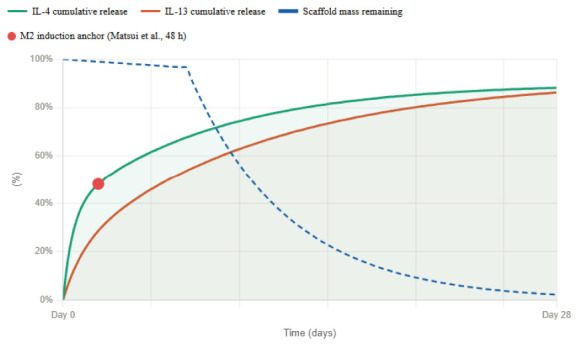
Degradation and release curve of Hydrogel III. illustrative mechanistic estimates based on studies (Miron et al., 2013; Fancy et al., 2009; Van Broeckhoven et al., 2021).

#### 4.4.3 Chemomodulators release

As explained in 2.1, following M2 macrophage polarization and efficient Wallerian debris clearance (Kigerl et al., 2009), the continuous, localized release of BDNF and NT-3 from the TEM prepares the spinal microenvironment for guided neurite growth (Cao and Shoichet, 2003; Song et al., 1997). These neurotrophins establish stable chemotactic gradients along the scaffold fibers, providing both trophic support and directional cues for regenerating axons (Blesch and Tuszynski, 2007; Song et al., 1997). By maintaining a permissive extracellular environment enriched with growth-promoting signals, BDNF and NT-3 ensure that neurites extend along the intended paths within the TEM, synergizing with the structural alignment of the hydrogel scaffold to maximize axonal guidance and functional regeneration (Cao and Shoichet, 2003; Han et al., 2009).

### 4.5 Glial scar removal via ABC chondroitinase

Chondroitinase ABC (ChABC) embedded in the TEM is gradually released into the lesion environment, enabling sustained digestion of inhibitory CSPGs (Bradbury et al., 2002). This passive, localized delivery maintains a permissive extracellular matrix, reducing glial scar barriers while the aligned TEM fibers and embedded neurotrophins guide regenerating axons. By combining enzymatic softening of the inhibitory ECM with structural and chemotactic cues, the continuous release of ChABC ensures that neurites encounter a growth-permissive corridor, supporting oriented axonal extension and preparing the microenvironment for the subsequent stages of targeted regeneration (Rossi et al., 2013).

Therefore, following the elimination of Wallerian debris, the combined presence of chemoattractants (BDNF and NT-3) and Chondroitinase ABC (ChABC) in the TEM establishes a permissive and directional microenvironment for regenerating neurites. The chemoattractants provide sustained trophic support and chemotactic gradients (Cao and Shoichet, 2003; Song et al., 1997), while ChABC digests inhibitory CSPGs, softening the extracellular matrix and reducing glial scar barriers. Importantly, these components function synergistically rather than antagonistically: the enzymatic activity of ChABC does not degrade the neurotrophins, and the trophic cues ensure that axons extend along the desired paths created by the aligned TEM scaffold (Massey et al., 2008). Together, they create a coordinated biochemical and structural corridor, primed to guide neurite growth efficiently, setting the foundation for the subsequent phases of targeted axonal regeneration.

### 4.6 Neuritic axonal growth

After the glial scar is removed, the pre-lesion axonal segment becomes exposed, and thanks to modulation of the spinal environment, it can extend neurites in the desired direction, guided by the previously administered chemoattractants (Song et al., 1997). This process eventually directs the axon through the tube formed by the TEM, guiding it along the original path of the post-lesion axonal segment toward its next target neuron. After the pre-lesion axon establishes synapses with the dendrites of the corresponding downstream neuron, the neuronal connection is physically restored, and the TEM is eventually naturally eliminated following the withdrawal of previously administered factors and agents (Han et al., 2009).

### 4.7 Axonal remyelination

Finally, the exposure of demyelinated axonal segments following TEM removal would promote oligodendrocyte migration and proliferation, ultimately leading to remyelination of the newly regenerated axonal portions (Miron et al., 2013). This process would restore the spinal environment to its pre-injury state, consolidating the newly formed structures (Nocera and Jacob, 2020).

## 5 Discussion and conclusion

This proposal represents an ambitious theoretical framework for spinal cord regeneration whose value lies primarily in its integrative logic rather than its immediate clinical applicability. By sequentially addressing the five principal barriers to CNS axonal regrowth within a single coherent protocol, it offers a conceptual architecture that much of the existing literature lacks. However, its limitations must be examined with the same rigor applied to its construction.

### 5.1 Speculative foundation and absence of direct experimental validation

No component of this protocol has been tested as part of the integrated sequence proposed here. Each individual intervention draws on studies conducted in isolation, typically in rodent models and often addressing partial rather than complete transection. The assumption that these interventions will behave synergistically when combined in a single protocol is not supported by direct evidence. Interaction effects between components remain entirely untested despite the compatibility arguments presented in section 2.2.4.

### 5.2 The hydrogel delivery problem

The most structurally critical limitation concerns the physical delivery of Hydrogel II and III across the full extent of the lesion. The human spinal cord lesion is a heterogeneous, dynamically evolving space that may span several centimeters rostrocaudally. A single injection point cannot produce homogeneous scaffold distribution, meaning both hydrogels would require stepwise microinjections at multiple levels to achieve continuous TEM formation. Each injection point introduces independent variability in gelation kinetics, fibrillar alignment, and neurotrophin gradient geometry. Similarly, focal Hydrogel III delivery risks leaving distal lesion regions with insufficient cytokine coverage, potentially sustaining M1 activity and debris accumulation in undertreated zones.

### 5.3 Temporal coordination and translational gap

The sequential logic of the protocol depends on precise temporal handovers between phases governed by biological processes not fully controllable in the clinical setting. In human SCI, the inflammatory trajectory is substantially more variable than in rodent models, and the subacute intervention window may close earlier or later than anticipated.

### 5.4 Value as a theoretical framework and next steps

Despite these limitations, the proposal makes a genuine conceptual contribution by demonstrating that the five barriers to CNS regeneration are causally interdependent, and that this interdependence logically determines a non-arbitrary sequence of intervention. This sequential logic is more rigorously derived than in most existing multi-modal SCI proposals, providing a structured basis for preclinical experimental design in which each phase can be tested independently before integration.

Importantly, the integrated protocol as proposed is sufficiently well-defined and mechanistically grounded to warrant formal preclinical testing in a murine complete spinal cord transection model. Such a study would constitute the necessary first step toward validating the sequential logic of the framework, establishing whether combined hydrogel delivery produces synergistic outcomes beyond individual components, and identifying which phases require refinement before scaling to larger models. A rigorous negative result would be as valuable as a positive one, as it would pinpoint the specific point of failure within the sequential chain.

In conclusion, this framework should be understood as a hypothesis-generating platform rather than a clinical protocol. Its logical coherence, combined with the individual evidence base supporting each component, makes it a credible candidate for structured preclinical investigation and a potentially valuable foundation for the next generation of translational SCI research.

## Data Availability

The original contributions presented in this study are included in this article/Supplementary materials, further inquiries can be directed to the corresponding author.
