# Supplementary material for: A sequential multimodal framework for spinal cord regeneration
Source: Front Cell Neurosci. 2026 Apr 29;20:1790692. doi: 10.3389/fncel.2026.1790692 (PMC13170459; doi:10.3389/fncel.2026.1790692)
Supplement: Supplementary file 1 [file Data_Sheet_1.pdf]

EXPERIMENTAL DESIGN PROPOSAL

Preclinical Validation of a Sequential Multimodal Framework for Spinal Cord Regeneration in a Murine Model

Eduardo Blat Sos

Universidad CEU Cardenal Herrera, Valencia, Spain

Supplementary Material — Frontiers in Cellular Neuroscience | Manuscript ID: 1790692

1. Experimental Objective

This experimental proposal is intended as a structured preclinical framework to illustrate the practical testability of the core falsifiable hypothesis advanced in the accompanying theoretical article, rather than as a finalized or optimized protocol. Specifically, it outlines a biologically coherent strategy to evaluate whether the temporally ordered, sequential application of: (1) localized immunomodulation, (2) mechanical realignment via microconnector, (3) a temporary extracellular matrix (TEM) incorporating chemoattractants and enzymatic CSPG degradation, and (4) M2-promoting Wallerian debris clearance, can promote directed axonal elongation and restore measurable functional connectivity in a murine model of acute spinal cord injury.

The central premise under evaluation is that the efficacy of each intervention depends not solely on its individual effect, but on its position within a defined temporal sequence. Accordingly, the proposed design is conceptually structured to compare the fully integrated protocol with partial implementations, allowing assessment of the relative contribution of each phase and of whether disruption of the sequence leads to predictable failure modes consistent with the underlying hypothesis.

2. Falsifiable Hypothesis

2.1 Null Hypothesis (H_0)

The complete sequential intervention — comprising localized DAMP-scavenging immunomodulation (Hydrogel I), microconnector implantation, TEM delivery with embedded chemoattractants and ChABC (Hydrogel II), M2-polarizing debris clearance (Hydrogel III), and sustained AAV-mediated ChABC expression — will produce no statistically significant improvement in axonal density, axonal length across the lesion, or functional motor recovery (BBB scale) compared to the untreated spinal cord injury control group (G0) at 8 weeks post-injury.

2.2 Alternative Hypothesis (H_1)

The complete sequential intervention (G4) will produce statistically significant increases in: (a) axonal density and oriented axonal length across the lesion site as quantified by GAP-43 and β-III tubulin immunohistochemistry; and (b) functional motor recovery as measured by BBB locomotor scale and horizontal ladder test — compared to G0 and to all partial treatment groups (G1, G2, G3). Furthermore, each successive addition of a protocol phase (G1 → G2 → G3 → G4) will produce a stepwise, monotonically increasing improvement in at least one primary outcome measure, reflecting the causal interdependency between sequential phases proposed in the theoretical framework.

This second prediction is what distinguishes the present hypothesis from a simple additive multimodal approach: it predicts a defined failure mode for each partial protocol, not merely a reduced benefit.

3. Experimental Model

3.1 Species and Model

Adult female mice (10–12 weeks, 20–25 g) will be used. The injury model will consist of a controlled T9–T10 thoracic transection) performed under isoflurane anesthesia (2–3%), with laminectomy exposing the T9 vertebral level. A microsurgical scalpel will be used to perform a complete, reproducible transection, confirmed by visual inspection and by the absence of hind-limb response upon dorsal root stimulation distal to the lesion. Post-operative analgesia (buprenorphine 0.05 mg/kg), bladder expression, and fluid support will be provided according to institutional animal care protocols.

3.2 Experimental Groups

A total of six groups will be established (n = 10 animals per group; n = 5 sacrificed at Week 4 for intermediate analysis, n = 5 at Week 8 for final endpoint), for a total of 60 animals:

Group	Name	Interventions
G0	Lesion Control	T9–T10 hemisection only; no implant, no hydrogel
G1	Microconnector	Lesion + microconnector implantation
G2	Microconnector + TEM	G1 + Hydrogel I (IL-10/PAMAM) + Hydrogel II (Collagen I / Laminin / RGD / BDNF / NT-3 / ChABC)
G3	Microconnector + TEM + Chemomodulators	G2 + Hydrogel III (IL-4 / IL-13) for Wallerian debris clearance
G4	Full Sequential Protocol	G3 + AAV-ChABC viral vector (sustained CSPG degradation): complete sequential intervention
GS	Sham	Laminectomy without SCI; all hydrogels administered to uninjured cord

All groups receive identical post-operative care. Randomization will be performed by block randomization prior to surgery. Investigators performing BBB assessments and histological quantification will be blinded to group assignment.

4. Sequential Intervention Protocol

The following protocol mirrors the theoretical sequence described in the article and is adapted for the murine spinal cord transection model. All steps are performed intraoperatively during the same surgical session unless otherwise specified.

4.1 Step-by-Step Surgical and Pharmacological Sequence

1. Laminectomy and lesion induction: T9–T10 laminectomy; controlled transection
2. Microsurgical debridement: Gentle removal of axonal debris, hematoma aspiration, and minimal coagulation to preserve viable parenchyma and reduce DAMP load.
3. Hydrogel I administration (G2–G4): Topical and intraparenchymal microinjection of IL-10/PAMAM hydrogel directly to the lesion cavity and surrounding inflammatory zone. Continued until inflammatory markers (monitored by ELISA sampling of lesion-adjacent tissue at scheduled sacrifices) decline to a pre-defined low-activity threshold (TNF-α < 50 pg/mL, IL-1β < 30 pg/mL).
4. Microconnector implantation (G1–G4): Under microscopic guidance, microconnector is placed to mechanically align proximal and distal cord stumps and establish a protected internal niche. Stumps are inserted into device channels and oriented parallel to the spinal axis.
5. Hydrogel II (TEM) administration (G2–G4): Stepwise intraparenchymal microinjection of the five-component TEM solution at 4°C; in situ gelation at physiological temperature. Volume and injection points determined by pre-operative MRI lesion volumetry. Delivery halted once lesion fill is confirmed by imaging.
6. AAV-ChABC injection (G4 only): Day 2–3 post-TEM, a single intraparenchymal injection of immune-evasive AAV5-ChABC is administered adjacent to the TEM site to initiate sustained in situ ChABC expression from Day 7–14 onward.
7. Hydrogel III administration (G3–G4): Once TEM integration is confirmed (Day 3–5), IL-4/IL-13 biodegradable hydrogel is administered. Cytokine delivery is tapered and scaffold undergoes complete biodegradation by Day 21, to prevent TGF-β-mediated fibrotic ECM remodeling.

4.2 Hydrogel Compositions and Delivery Parameters

Hydrogel	Composition	Timing	Objective
Hydrogel I	IL-10 (12.5 µg/mL) + PAMAM-G3 in photocrosslinked gelatin	Immediately post-debridement; biodegrades by ~8 weeks	DAMP scavenging; M2 macrophage polarization; anti-inflammatory microenvironment prior to TEM
Hydrogel II (TEM)	Collagen I (0.6–0.8 mg/mL) + Laminin + RGD peptides + BDNF + NT-3 + ChABC (trehalose-stabilized)	After Hydrogel I stabilization; intraparenchymal microinjection	Structural scaffold; axonal contact guidance; chemoattractant gradients; CSPG degradation (protein phase)
Hydrogel III	IL-4 (10 ng/mL) + IL-13 in biodegradable carrier	Once TEM integration confirmed (~Day 3–5); tapered Days 7–21	M2 polarization for Wallerian debris clearance; remyelination priming via activin-A
AAV-ChABC (G4 only)	Immune-evasive AAV5/AAV9 encoding ChABC; single intraparenchymal injection	Day 2–3 post-TEM; expression onset Day 7–14; sustained ≥12 weeks	Sustained in situ CSPG degradation beyond protein-phase window; seamless handover from Hydrogel II

5. Outcome Measures and Evaluations

Category	Measure	Expected Outcome (G4 vs G0)
Functional – Motor	BBB Locomotor Scale (0–21)	Significantly higher BBB scores in G4 vs G0/G1 at weeks 6–8
Functional – Motor	Horizontal Ladder Test (foot fault %)	Reduced foot fault rate in G4 indicating improved stepping accuracy
Histology – Axonal	GAP-43 and β-III tubulin IHC (axon density & length)	Increased axon density and length across lesion site in G4
Histology – Glial	GFAP (astrocytes) and Iba1 (microglia) IHC	Reduced astrogliosis and microglial activation in G4 vs G0
Histology – Matrix	CS-56 IHC (CSPG quantification)	Markedly reduced CSPGs in G4; partial reduction in G2/G3
Morphometry	Axon count, length, orientation angle, cavity volume	G4 shows directed elongation perpendicular to lesion; minimal cavity formation
Inflammatory Profile	ELISA: IL-1β, TNF-α, IL-10, TGF-β at Days 3, 7, 21	Sustained low IL-1β/TNF-α in G4; elevated IL-10 at Day 3 declining by Day 21
Biocompatibility	Tissue reaction to biomaterials; H&E morphology	No adverse foreign body response; normal tissue integration in all hydrogel groups

5.1 Functional Evaluations

BBB Locomotor Scale assessments will be performed weekly from Day 1 post-injury through Week 8 by two independent blinded observers. Scores will be averaged per animal per timepoint. The horizontal ladder test (10 trials per session) will be performed at Weeks 2, 4, 6, and 8, with foot fault percentage calculated as the proportion of hindlimb steps resulting in a foot fault.

5.2 Histological Evaluations

At sacrifice, animals will be transcardially perfused with PBS followed by 4% paraformaldehyde. Spinal cords will be extracted, post-fixed, cryoprotected in 30% sucrose, and sectioned longitudinally at 20 μm on a cryostat. The following markers will be assessed by immunofluorescence:

- GAP-43 and β-III tubulin: axonal regeneration markers (density and oriented length)
- GFAP: reactive astrocyte quantification (glial scar extent)
- Iba1: microglial/macrophage activation
- CS-56: chondroitin sulfate proteoglycan (CSPG) residual load
- MBP (myelin basic protein): remyelination assessment at Week 8

5.3 Morphometric Analysis

Axonal quantification will be performed using confocal microscopy with automated image analysis (ImageJ/FIJI with NeuronJ plugin). Parameters measured will include: total axon count across the lesion midpoint, mean axon length, axonal orientation angle relative to the rostrocaudal axis (as a measure of directed elongation), and residual cavity volume by volumetric reconstruction.

5.4 Inflammatory and Biocompatibility Profile

At intermediate sacrifice (Week 4), a 3 mm segment of spinal cord centered on the lesion will be homogenized for ELISA quantification of IL-1β, TNF-α, IL-10, and TGF-β. Biocompatibility will be evaluated by H&E morphology of tissue surrounding all implanted materials.

6. Study Design, Timeline, and Statistical Analysis

6.1 Sample Size Justification

Sample size of n = 10 per group (5 per timepoint) is based on published SCI motor recovery studies using the BBB scale, which report effect sizes of 0.8–1.2 between treatment and control groups at 8 weeks (power = 0.80, $\alpha = 0.05$, two-tailed). This yields a minimum n = 8 per group; n = 10 is used to account for expected attrition of ~15–20% in complete hemisection models.

6.2 Statistical Analysis

Primary outcomes (BBB score, axon density, CSPG levels) will be analyzed using one-way ANOVA with post-hoc Tukey correction for multiple comparisons across groups at each timepoint. Repeated-measures ANOVA will be applied to longitudinal BBB data. The stepwise hypothesis (monotonic improvement $G0 < G1 < G2 < G3 < G4$) will be tested using a linear contrast analysis. All statistical analyses will be performed with $\alpha = 0.05$ as the significance threshold. All data will be presented as mean ± SEM.

7. Predicted Outcomes and Failure Mode Analysis

A critical feature of this experimental design — one that distinguishes it from standard multimodal SCI studies — is the prediction of specific failure modes for each partial protocol group. These predictions follow directly from the causal logic of the sequential framework and provide falsifiable benchmarks against which each phase's necessity can be assessed:

- G1 (Microconnector only): Physical alignment without anti-inflammatory conditioning is predicted to fail at TEM integration. Histology should show poor scaffold adhesion and sustained M1 macrophage dominance at the lesion interface.
- G2 (Microconnector + TEM): Without M2-polarizing Wallerian debris clearance, persistent myelin inhibitors are predicted to re-convert M2 macrophages toward M1 (TLR2/4 re-activation), stalling neurite extension before 500 μm and producing disorganized GAP-43 immunoreactivity.
- G3 (Microconnector + TEM + Wallerian clearance): Without sustained AAV-mediated ChABC delivery, protein-phase CSPG degradation will decline after Week 4, predicted to produce a 'stalling zone' where axons reach but do not cross the distal scar margin. CS-56 immunoreactivity should show partial clearance centrally but persistent CSPG accumulation at the lesion periphery.
- G4 (Full protocol): Predicted to produce the highest BBB scores, greatest axon density across the lesion, lowest residual CSPG load, and directed axonal orientation consistent with TEM guidance. This group constitutes the primary test of the integrated hypothesis.
- GS (Sham): All hydrogel components administered to uninjured cord should produce no adverse tissue reaction, confirming biocompatibility in the absence of pathological microenvironment. Any tissue reaction in GS would indicate material-specific toxicity rather than procedure-dependent effects.

8. Anticipated Limitations and Contingency Plans

The following limitations are acknowledged and addressed:

- Heterogeneity of TEM distribution: The injured murine hemicord lesion, though smaller than the human SCI lesion, may still produce non-uniform hydrogel fill. Multiple microinjection coordinates (rostral, central, caudal to lesion midpoint) will be used to maximize homogeneous coverage. Post-injection MRI will verify distribution before proceeding to subsequent steps.
- Temporal variability in inflammatory clearance: The threshold criterion for Hydrogel I discontinuation (TNF-α < 50 pg/mL) will be monitored using a parallel cohort of 2 animals per group sacrificed at Day 3 for ELISA validation. If inflammatory clearance is slower than anticipated, the Hydrogel I administration window will be extended accordingly.
- AAV transduction efficiency: AAV5-ChABC expression onset and titer will be confirmed in a pilot cohort of 3 animals prior to the main study, using western blot for ChABC protein and CS-56 depletion at Day 14.

9. Study Duration

Total follow-up: 8 weeks post-injury. Intermediate histological endpoint at Week 4 (n = 5 animals per group sacrificed). Final endpoint at Week 8 (remaining n = 5 per group). Weekly behavioral assessments from Day 1 to Week 8. ELISA profiling at Days 3, 7, 21, and final sacrifice.

Estimated total study duration from surgery to data analysis: 5–6 months, including pilot validation of AAV titer and hydrogel distribution parameters prior to full cohort enrollment.
